# Diet-Derived Advanced Glycation End-Products (AGEs) Induce Muscle Wasting In Vitro, and a Standardized *Vaccinium macrocarpon* Extract Restrains AGE Formation and AGE-Dependent C2C12 Myotube Atrophy

**DOI:** 10.3390/antiox14080900

**Published:** 2025-07-23

**Authors:** Martina Paiella, Tommaso Raiteri, Simone Reano, Dominga Manfredelli, Tommaso Manenti, Giulia Gentili, Hajar Meskine, Sara Chiappalupi, Giovanni Bellomo, Flavia Prodam, Cinzia Antognelli, Roccaldo Sardella, Anna Migni, Guglielmo Sorci, Laura Salvadori, Nicoletta Filigheddu, Francesca Riuzzi

**Affiliations:** 1 Department of Translational Medicine, University of Piemonte Orientale, 28100 Novara, Italy; martina.9702@gmail.com (M.P.); simone.reano@uniupo.it (S.R.); hajar.meskine@uniupo.it (H.M.); laura.salvadori@uniupo.it (L.S.); nicoletta.filigheddu@med.uniupo.it (N.F.); 2 Interuniversity Institute of Myology (IIM), 06132 Perugia, Italy; tommaso.raiteri@unipg.it (T.R.); giuliagentili18@gmail.com (G.G.); sarac.chiappalupi@gmail.com (S.C.); guglielmo.sorci@unipg.it (G.S.); 3 Department of Medicine and Surgery, University of Perugia, 06123 Perugia, Italy; dominga.manfredelli@dottorandi.unipg.it (D.M.); giovanni.bellomo@unipg.it (G.B.); cinzia.antognelli@unipg.it (C.A.); 4 Laboratori Biokyma Srl, 52031 Anghiari, Italy; tmanenti@biokyma.com; 5 Department of Health Sciences, University of Piemonte Orientale, 28100 Novara, Italy; flavia.prodam@med.uniupo.it; 6 Department of Pharmaceutical Sciences, University of Perugia, 06123 Perugia, Italy; roccaldo.sardella@unipg.it (R.S.); anna.migni@dottorandi.unipg.it (A.M.)

**Keywords:** diet-derived AGEs, glycated albumin, RAGE, ROS production, muscle atrophy, natural compounds, mitochondrial function

## Abstract

Dietary advanced glycation end-products (dAGEs) contained in high-sugar/fat and ultra-processed foods of the “Western diet” (WD) pattern predispose to several diseases by altering protein function or increasing oxidative stress and inflammation via RAGE (receptor for advanced glycation end-products). Although elevated endogenous AGEs are associated with loss of muscle mass and functionality (i.e., muscle wasting; MW), the impact of dAGEs on MW has not been elucidated. Here, we show that the most common dAGEs or their precursor, methylglyoxal (MGO), induce C2C12 myotube atrophy as endogenous AGE-derived BSA. ROS production, mitochondrial dysfunction, mitophagy, ubiquitin–proteasome activation, and inhibition of myogenic potential are common atrophying mechanisms used by MGO and AGE-BSA. Although of different origins, ROS are mainly responsible for AGE-induced myotube atrophy. However, while AGE-BSA activates the RAGE-myogenin axis, reduces anabolic mTOR, and causes mitochondrial damage, MGO induces glycolytic stress and STAT3 activation without affecting RAGE expression. Among thirty selected natural compounds, *Vaccinium macrocarpon* (*VM*), *Camellia sinensis*, and chlorophyll showed a surprising ability in counteracting in vitro AGE formation. However, only the standardized *VM*, containing anti-glycative metabolites as revealed by UHPLC-HRMS analysis, abrogates AGE-induced myotube atrophy. Collectively, our data suggest that WD-linked dAGE consumption predisposes to MW, which might be restricted by *VM* food supplements.

## 1. Introduction

Skeletal muscle tissue accounts for 45–50% of total human body mass and is fundamental for several body functions, playing a crucial role in locomotion, respiration, energy metabolism, and substrate turnover and storage [1,2]. Muscle homeostasis perturbations can have significant metabolic consequences, predisposing to several diseases [2]. Skeletal muscle wasting (MW), characterized by a loss of muscle mass (atrophy) and strength, is a common feature contributing to harmful outcomes of noncommunicable chronic diseases (NCDs), such as diabetes, obesity, and sarcopenia (i.e., the specific MW naturally occurring with aging) [3]. MW originates from the interconnection of multiple mechanisms that eventually result in an imbalance between protein synthesis and degradation in favor of the latter. Oxidative stress, arising both from endogenous and exogenous sources, plays a central role in MW as it can increase muscle catabolism by activating both the ubiquitin–proteasome system (UPS) and the autophagy-lysosomal system (ALS), the two major pathways responsible for muscle atrophy, as well as decreasing protein synthesis rate impinging on the AKT-mTOR pathway [4]. Furthermore, oxidative stress may impair muscle regenerative potential by inhibiting the myogenic differentiation of satellite cells, the adult stem cells of muscle tissue, thus contributing to the loss of skeletal muscle mass, especially during aging [5]. Mitochondria are the main source of reactive oxygen species (ROS) in skeletal muscle due to physiological cellular metabolism and contribute to the oxidative stress when their function is compromised. Pathophysiological conditions (including inflammation, metabolic disorders, tumors, and aging) and lifestyle factors (including smoking and a poor diet) can induce mitochondrial dysfunction and oxidative stress [6].

In recent decades, a nutritional transition to a “Western diet” (WD), characterized by a high intake of low-quality ultra-processed foods rich in sugars and saturated fats and poor in fruits, vegetables, and fibers, has contributed to the global exacerbation of NCDs. Indeed, WD contributes to the development of insulin resistance and metabolic inflexibility by triggering inflammation and oxidative stress [7]. The advanced glycation end-products (AGEs) contained in WD foods are considered one of the molecular mediators in the onset and progression of metabolic dysfunctions [8]. AGEs represent a heterogeneous group of non-enzymatic adducts between reducing sugars and the free amino groups of proteins, nucleic acids, and lipids, usually resulting in fluorescent derivatives [9]. Endogenous AGE formation, principally glycated albumin, occurs in conditions of hyperglycemia and oxidative stress and during aging, whereas exogenous AGEs (i.e., diet-derived AGEs (dAGEs)) are derived from the consumption of WD, especially saturated fats, sugars, meat, and cheese [9,10]. The preparation methods (heat and dehydration) and processing (grilling, frying) typical of WD induce further generation of dAGEs, which the gastrointestinal tract can absorb and are only partially excreted in the urine, with a significant accumulation in various tissues [10,11]. Methylglyoxal (MGO) is a potent precursor of fluorescent AGEs, such as 5-hydro-5-methylimidazolone (MG-H1) and carboxymethyl-L-lysine (CML), and the non-fluorescent AGE, pentosidine (PENT), which are widely used as markers of dAGE accumulation in human tissues [9,11]. AGEs may directly alter the structure and function of tissue cross-linked proteins and may induce tissue injury, leading to the generation of ROS and amplifying inflammation by interacting with their receptor, RAGE (receptor for advanced glycation end-products) [9]. The correlation between AGE accumulation/activity and MW is supported by substantial evidence [12]. Specifically, (i) high AGE levels in skeletal muscle, blood, and skin are associated with sarcopenia in diabetic patients and elderly subjects [13,14]; (ii) mice fed with an AGE-enriched diet exhibit high levels of CML in muscles, along with reduced muscle mass and endurance [15]; (iii) fructose-derived AGEs activate metabolic reprogramming and mitochondrial dysfunctions in muscles [16]; and (iv) glycated albumin (AGE-BSA) induces atrophy in C2C12 and human myotubes by activating the UPS via RAGE [17]. Similarly, lifelong and short-term WD consumption accelerates age-associated muscle decline [18] and exacerbates denervation-induced MW by impairing mitochondrial function [19], respectively. In contrast with WD, adherence to the Mediterranean dietary pattern is positively associated with handgrip strength and appendicular skeletal muscle mass in the elderly [20].

Since the direct role of dAGEs in the onset of MW has not been investigated so far, we tested the effects of different dAGEs on myotubes in vitro in comparison with the endogenous AGE-BSA. We found that dAGEs induce myotube atrophy by activating the catabolic program and affecting mitochondrial functionality, although using different molecular mechanisms from AGE-BSA.

Natural active metabolites, including polyphenols, polysaccharides, terpenoids, and vitamins, have recently appeared as promising agents to reduce the deleterious accumulation/activity of AGEs in many diseases, including NCDs [21]. These metabolites have several advantages over anti-glycation synthetic compounds, which are associated with severe side effects in humans [22]. Thus, we tested several natural compounds (i.e., standardized dry extracts from twenty-one officinal plants and four mushrooms) and five active compounds, based on their proven activity as antioxidant and/or anti-inflammatory agents or on their ethnobotanical use to prevent or reduce the accumulation/activity of dAGEs, for their protection against dAGE-induced myotube atrophy.

## 2. Materials and Methods

### 2.1. Endogenous and dAGEs

AGE-BSA was obtained by adding 1 g of BSA and 1.8 g of D-glucose in 20 mL of PBS. The solution was aliquoted at a 50 mg/mL concentration and stored at −80 °C [23]. MGO solution (M0252) was purchased from Sigma-Aldrich (St. Louis, MO, USA), CML (CAS 5746-04-3) from Santa Cruz Biotechnology (Dallas, TX, USA), and pentosidine (10010254) from Cayman Chemical (Ann Arbor, MI, USA).

### 2.2. Cell Cultures and Treatments

Murine C2C12 myoblasts were cultured in Dulbecco’s Modified Eagle’s Medium (DMEM) containing high glucose (4500 mg/L) and supplemented with 20% fetal bovine serum (FBS), 100 U/mL penicillin, and 100 mg/mL streptomycin (P/S) (growth medium, GM). Myotubes were obtained by shifting sub-confluent myoblasts to DMEM supplemented with 2% horse serum (HS) (differentiation medium, DM) for 4 days [24]. AGE-BSA (50–800 µg/mL), MGO (100–1000 µM), CML (100–800 µM), or PENT (1–8 µM) in combination or not with *V. macrocarpon* (*VM*) were added to myotubes (25–1000 µg/mL) or myoblasts (100 µg/mL) for the indicated times. To investigate the role of oxidative stress, the antioxidants N-Acetyl-L-Cysteine (NAC; CAS 616-91-1; Thermo Fisher Scientific, Waltham, MA, USA) at 5 mM and MitoTEMPO (CAS 1334850-99-5; Sigma-Aldrich) at 10 µM were added during the final 24 h of treatment with AGEs.

### 2.3. May–Grünwald/Giemsa Staining

The cells were fixed and processed as previously described [24]. The samples were acquired (Olympus IX51, Tokyo, Japan) at 4× magnification, and myotube areas were measured in each photo using ImageJ software (v. 1.53j, National Institutes of Health, Bethesda, MD, USA).

### 2.4. Western Blotting

Myotube cultures were lysed in protein extraction buffer [24]. Equal amounts of total protein extract (20 to 30 µg) were resolved by SDS-PAGE (Sodium Dodecyl Sulfate–Poly Acrylamide Gel Electrophoresis) and transferred to nitrocellulose blots (Amersham™ Protran^®^, 0.45 µm; Wilmington, DE, USA). Following blocking with 5% nonfat dry milk or Roti-Block, primary and secondary antibodies were applied as indicated in Table S1. The immune reactions were developed by enhanced chemiluminescence. C-DiGit Blot Scanner (LI-COR, Lincoln, NE, USA) or Ibright CL1500 System (Thermo Fisher Scientific, Waltham, MA, USA) were used for blot analysis. Protein sizes were estimated with prestained protein ladders from Thermo Fisher Scientific or ABclonal (Woburn, MA, USA).

### 2.5. Real-Time PCR

RNA extraction, reverse transcription, and real-time PCR analyses of mRNA contents were performed as previously described [24,25]. The calculation was performed using software MXPRO-Mx 3000P v. 4.10 (Agilent Technologies, Santa Clara, CA, USA) in comparison with a standard gene (*Gapdh*). The primers are reported in Table S2. For *Opa1* (Mm00453873_m1), *Mff* (Mm01273401_m1), *Bnip3* (Mm01275600_g1), *Gabarap* (Mm00490678_m1), *Atg12* (Mm00503201_m1), *Pgc1a* (Mm01208835_m1), *Mnf2* (Mm00500120_m1), and *Atf4* (Mm00515325_g1) genes, RNA was retro-transcribed with the High-Capacity cDNA Reverse Transcription Kit (Applied Biosystems, Thermo Fisher Scientific, Waltham, MA, USA), and real-time PCR was performed using the StepOnePlus Real-time PCR System (Applied Biosystems, Thermo Fisher Scientific) using the TaqMan probes (Thermo Fisher Scientific). *Gusb* (Mm01197698_m1) was used as the housekeeping gene.

### 2.6. Cell Fractionation

At the end of the indicated treatments, cells were harvested by trypsinization, resuspended in ice-cold fractionation buffer (20 mM HEPES, 10 mM KCl, 2 mM MgCl_2_, 1 mM EDTA, 1 mM EGTA, pH 7.4) supplemented with protease inhibitor cocktail, and incubated for 15 min on ice. Then, cells were mechanically lysed by repeated passage through a 29-gauge needle and incubated for an additional 20 min on ice. Afterwards, lysates were centrifuged at 720× *g* for 5 min at 4 °C to remove nuclei and unbroken cells. The resulting supernatant was centrifuged at 10,000× *g* for 5 min at 4 °C to pellet the mitochondrial fraction. The pellet was resuspended in TBS containing 0.1% SDS and sonicated to obtain the mitochondrial lysate.

The mitochondrial fractions were subsequently analyzed by Western blotting.

### 2.7. Intact Cell Respiration Using High-Resolution Respirometry

Cellular respiration was measured using an Oroboros oxygraph-2K high-resolution respirometer (Oroboros Instruments GmbH, Innsbruck, Austria) and the “substrate, uncoupler, inhibitor, titration” (SUIT) protocol SUIT-003_O2_ce_D012, as recommended by the manufacturer of the instrument and as previously described [25]. Briefly, at the end of the treatments, C2C12 myotubes were trypsinized, centrifuged at 300× *g* for 5 min, resuspended in the mitochondrial respiration medium MiR05 (0.5 mM EGTA, 3.0 mM MgCl_2_·6H_2_O, 60 mM potassium lactobionate, 20 mM taurine, 10 mM KH_2_PO_4_, 20 mM HEPES, 110 mM sucrose, 1 g/L BSA, pH 7.1), and transferred to the chambers of the Oroboros oxygraph. Control and treated samples were assessed simultaneously. After the initial stabilization of O_2_ flux, 5 mM pyruvate was used to sustain TCA-linked respiration in the MiR05 medium. The ATP synthetase inhibitor oligomycin (Omy) was added at the concentration of 5 nM, and the oligomycin-sensitive and -insensitive respiration was determined by quantifying oxygen consumption. Carbonyl cyanide-p-trifluoromethoxyphenylhydrazone (FCCP), a protonophore and uncoupler of oxidative phosphorylation, was then added at 0.5 µM increments to achieve maximum respiration to quantify the maximal respiratory capacity. Finally, the inoculation of 500 nM rotenone (Rot) followed by 2.5 µM antimycin A (Ama) to inhibit complex I and III of the ETS, respectively, was used to determine the non-mitochondrial respiration (ROX). The rates of O_2_ consumption (flux), calculated with the software DatLab v.7 (Oroboros Instruments GmbH, Innsbruck, Austria), were normalized to the total protein content obtained at the end of the experimental procedure by centrifuging at 1000× *g* for 5 min the cellular suspension from the two chambers, lysing the cellular pellet in 200 µL of lysis buffer (10 mM HEPES, 60 mM KCl, 1 mM EDTA, 0.075% NP40, 1 mM DTT), and then centrifuging the lysates at 15,000× *g* for 15 min at 4 °C. The concentration of the protein in the supernatant was measured with the Bradford Reagent (Thermo Fisher Scientific).

### 2.8. Quantification of ROS Production

To measure ROS production, C2C12 myotubes were stained with CellROX R Deep Red Reagent (Thermo Fisher Scientific) for 30 min at 37° C and washed with PBS. Fluorescent images were taken using a fluorescence microscope EVOSTM XL (Thermo Fisher Scientific, Waltham, MA, USA), and the mean fluorescence signal intensity was measured using ImageJ (v. 1.53j, National Institutes of Health, Bethesda, MD, USA). For every experiment assessing cellular oxidative stress, at least five myotubes in each field, five different fields for each replicate, and three technical replicates for each treatment were measured. The final data are the average of three independent experiments.

### 2.9. Natural Compounds

Standardized dried extracts of officinal plants (Table 1) and mushrooms (Table 2), and active principles (Table 3) were provided by Laboratori Biokyma S.r.l., Anghiari (AR), Italy. The extracts were from A.C.E.F. Srl (Fiorenzuola d’Arda, PC, Italy). The same batch of products was used in the various experiments. The compounds were solubilized in water at a concentration of 10 mg/mL and then appropriately diluted to be tested at the final concentrations reported in the specific bioassays.

### 2.10. Albumin Glycation Assay Kit

The Albumin Glycation Assay Kit [Glyceraldehyde] (catalog number AAS-AGE-K01E; Cosmo Bio, Carlsbad, CA, USA) consists of an inhibition assay for glycation of albumin formation by glyceraldehyde. The ability of natural compounds to inhibit glyceraldehyde-derived AGEs was tested in comparison with anti-glycation standard aminoguanidine, following the manufacturer’s instructions.

### 2.11. Cell Viability Assay

C2C12 myotubes were treated with *VM*, chlorophyll (CL), and *C. sinensis* (*CS*) (100–1000 µg/mL) for 24 h and incubated with 110 µL of medium containing MTT (3-[4,5-dimethylthiazol-2-yl]-2,5-diphenyl tetrazolium bromide; Sigma-Aldrich), 50 µg. After 4 h, the solubilization buffer (SDS 10% in HCl 0.01 M) was added to each well, and absorbance at 570 nm was measured using a UV/visible spectrophotometer (TECAN, Morrisville, NC, USA).

### 2.12. Ultra-High Performance Liquid Chromatography–Tandem Mass Spectrometry (UHPLC-HRMS/MS) Analysis

Liquid chromatographic separation and mass spectrometric analysis were performed on a UHPLC-MS/MS system consisting of an Agilent 1290 Infinity II combined with the Agilent 6560 ion mobility Q-TOF mass spectrometer (Agilent Technologies Inc., Santa Clara, CA, USA). The chromatographic separation was performed using a ZORBAX Rapid Resolution HD Eclipse Plus C18 column (50 mm × 2.1 mm, 1.8 µm, 95 Å, Agilent Technologies Inc., USA). UHPLC eluents A and B were, respectively, water and acetonitrile (LC-MS grade, LiChrosolv, Supelco, Merk KGaA, Darmstadt, Germany), both with 0.1% (*v*/*v*) formic acid (LC-MS grade, LiChropur, Supelco). The optimized gradient program was the following: 0–2.5 min, 5% (*v*/*v*) B; 2.5–4 min, 5–25% (*v*/*v*) B; 4–7 min, 25–35% (*v*/*v*) B; 7–9.5 min, 35–97% (*v*/*v*) B; 9.5–10.5 min, 97% (*v*/*v*) B; 10.5–11 min, 97–5% (*v*/*v*) B; 11–16 min, 5% (*v*/*v*) B (column equilibration/conditioning). The column temperature was set at 30 °C and the flow rate at 0.3 mL min^−1^. The injection volume was 5 µL. For MS detection, the Dual AJS ESI source operated in both positive and negative ion modes (https://www.sciencedirect.com/topics/chemistry/positive-ion; accessed on 5 May 2025). The gas temperature was set at 300 °C with a flow of 5 L min^−1^, while the sheath gas temperature was 350 °C with a flow of 11 L min^−1^. The nebulizer pressure was set at 35 psi, and the capillary and fragmentor voltages were 3000 V and 200 V, respectively. MS^2^ analysis was carried out using iterative automated (auto)-MS/MS acquisition in both ionization modes. In this case, the fragmentation patterns of the compounds were recorded at a fixed collision energy (20 eV) with an isolation width of 4 *m*/*z*. The Masshunter Workstation Data Acquisition 10.0 (Agilent Technologies Inc.) program was used for data acquisition, whereas the Masshunter Qualitative Analysis 10.0 (Agilent Technologies Inc.) software was used for data processing. To perform the analyses, 10 mg of dried *VM* extract powder was solubilized in a 15 mL glass tube with 1 mL of water and filtered using 0.22 µm nylon membrane filters and then diluted to achieve the final concentration of 10 ng/µL.

### 2.13. Immunofluorescence (IF) and Myotube Diameter Measure

For MyHC-II staining, myotubes were fixed with 4% paraformaldehyde (PFA), permeabilized using 0.1% Triton X-100 in PBS, blocked with blocking buffer containing 1% glycine (SERVA, Heidelberg, Germany) and 3% bovine serum albumin (BSA, Sigma-Aldrich) in PBS, and incubated in a humid chamber overnight at 4 °C with mouse anti-MyHC-II primary antibody (eBiosciences, San Diego, CA, USA) in PBS containing 3% BSA. The next day, coverslips were incubated with anti-mouse Alexa Fluor 488-conjugated antibody (Sigma-Aldrich) in PBS containing 3% BSA in a light-tight humid chamber. Nuclei were counterstained with DAPI (4′,6-diamidino-2-fenilindolo diidrocloruro; Sigma-Aldrich). Samples were mounted with a fluorescent mounting medium and viewed by an epifluorescence microscope (Leica DMRB, Milan, Italy) equipped with a digital camera. Myotube diameters were determined on images of MyHC-II staining at 20× magnification using ImageJ software as previously described [24]. Average diameters of at least 100 myotubes from 10 randomly chosen fields for each condition were determined. The width of each myotube was measured at 3 different points along the longitudinal axis of the cell.

### 2.14. Morphometric Evaluations

The fusion index (FI) was calculated as a percentage of nuclei in myotubes containing a minimum of 3 nuclei/total nuclei in 5 randomly selected fields per well after May–Grünwald/Giemsa staining at 10× magnification. Nuclei per myotube (NpM) were counted in 50 randomly chosen myotubes after May–Grünwald/Giemsa staining at 10× magnification [24].

### 2.15. BrdU Assay

For 5-bromo-2′-deoxyuridine (BrdU) staining, C2C12 myoblasts were seeded on pretreated glass coverslips at 5 × 10^4^ cells/well, and the day after, they were switched to DM in the presence of *VM* (100 µg/mL). After 24 h, BrdU (10 µM) was added for 1 h, and the cells were fixed in cold absolute methanol for 10 min and permeabilized for 5 min with 0.1% Triton X-100 in PBS. Samples were incubated with hydrochloric acid (HCl, 2N) for 30 min and washed with borate buffer (0.1 M, pH 8.3) followed by PBS before incubation with a mouse monoclonal anti-BrdU primary antibody (1:50; Santa Cruz Biotech, Dallas, TX, USA) in 3% BSA in PBS for 1 h at room temperature (RT). After washes in T-PBS and PBS, samples were incubated with the secondary anti-mouse Alexa Fluor 488-conjugated antibody (1:100 in T-PBS; Sigma-Aldrich) for 1 h at RT. Nuclei were counterstained with DAPI. Samples were mounted with a fluorescent mounting medium and viewed by an epifluorescence microscope (Leica DMRB, Milan, Italy) equipped with a digital camera.

### 2.16. MG-H1 and Glo1 Detection

MG-H1 was measured either by the OxiSelectTM methylglyoxal competitive enzyme-linked immunosorbent assay (ELISA) kit (DBA Italia Srl, Segrate, Italy) according to the manufacturer’s instructions. Glo1 activity was measured as previously described [26]. Shortly, the assay solution contained 0.1 M sodium phosphate buffer (pH 7.2), 2 mM Mg, and 1 mM GSH. The reaction was monitored spectrophotometrically by following the increase in absorbance at 240 nm and 25 °C. One unit of enzyme activity was defined as 1 µmol of S-D-lactoylglutathione per minute. Glo1-specific enzyme activity was calculated by relating Glo1 enzyme activity to the amount of total protein, which was determined by the Lowry method.

### 2.17. Statistical Analysis

Unless otherwise specified, data are presented as means ± SD (standard deviation) or SEM (standard error of the mean) of three independent experiments. Counts were performed by three independent operators blind to the treatments. Outliers in the measurements were identified by means of the interquartile range (IQR) as either below Q1 − 1.5 IQR or above Q3 + 1.5 IQR and excluded from the analysis. Representative experiments and images are shown. The variation among groups was evaluated using a one-way ANOVA test followed by Tukey’s multiple-comparisons test; *p* values < 0.05 were considered statistically significant.

## 3. Results

### 3.1. Dietary AGEs and AGE-BSA Reduce Myotube Areas and Induce MyHC-II Protein Degradation Through Only Partially Overlapping Mechanisms

To investigate the atrophying potential of exogenous AGE vs. the most common endogenous AGE, glucose-modified albumin, we tested two preformed AGEs (i.e., CML and PENT) and MGO as an AGE precursor, since they are characterized by different chemical properties and are contained in different foods. In particular, glyoxal-derived CML is a non-fluorescent and non-crosslinked AGE formed on protein by combining nonenzymatic glycation and oxidation reactions (glycoxidation). CML is the most used marker for AGE detection in unprocessed and processed food products [10]. PENT results from the reaction of pentose with lysine and arginine, especially during heating and storage of food, and is a fluorescent and protein-crosslinked AGE [27]. MGO is a highly reactive dicarbonyl compound generated during the Maillard reaction. This is a potent glycating agent and a predominant precursor of a heterogeneous family of final MGO-AGEs [28], the major one of which is MG-H1 [29]. MGO is ubiquitously present in foods rich in carbohydrates and fats and in fermented beverages [28].

**Figure 1:**
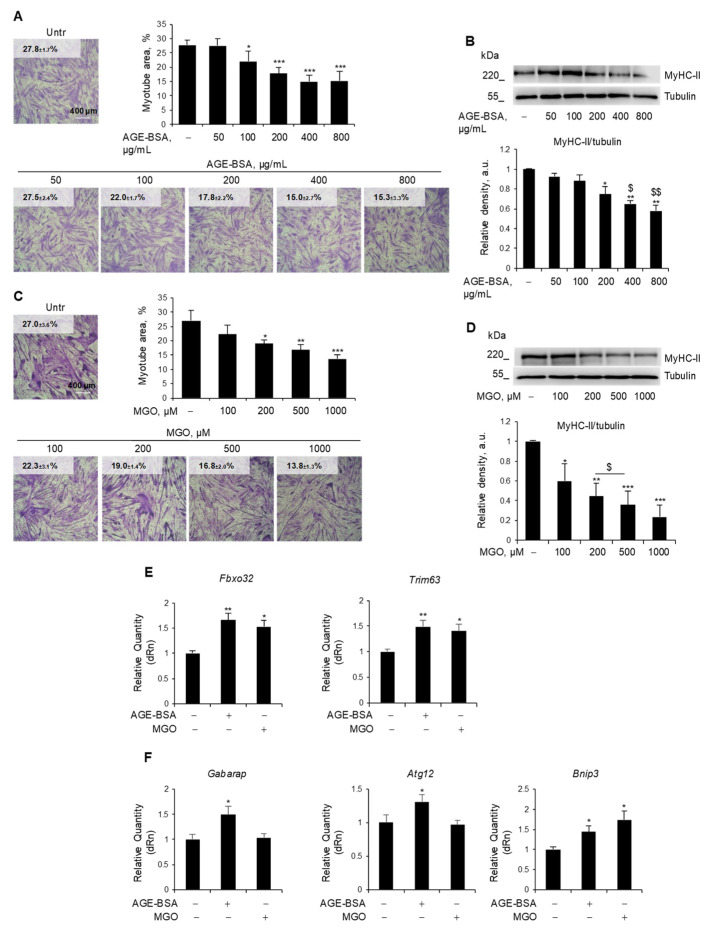
Endogenous and exogenous AGEs reduced myotube area and MyHC-II expression via partially distinct mechanisms. (**A**–**F**) C2C12 myotubes obtained from myoblasts cultured in differentiation medium (DM) for 4 days were treated with different doses of AGE-BSA (50–800 µg/mL) or the precursor of dietary AGEs, methylglyoxal (MGO; 100–1000 µM), for 48 h (**A**–**D**) or 24 h (**E**,**F**). Myotube areas were measured using ImageJ software after May–Grünwald/Giemsa staining. Reported are the percentages of myotube area (**A**,**C**). Myosin heavy chain (MyHC)-II expression was evaluated by Western blotting (WB) analysis, and the relative densities with respect to tubulin were determined (**B**,**D**). The expression of the atrogenes *Fbxo32* and *Trim63* (**E**) and the expression of the autophagy-related genes *Gabarap*, *Atg12*, and *Bnip3* (**F**) were assessed by real-time PCR, using *Gapdh* or *Gusb* as housekeeping genes (**F**). Representative images were reported (**A**–**D**). Data are means ± SEM (**A**,**C**) or SD (**C**,**D**–**F**) of three independent experiments. Statistical analysis was conducted using one-way ANOVA. * *p* < 0.05, ** *p* < 0.01, and *** *p* < 0.001, significantly different from Untr; ^$^ *p* < 0.05 and ^$$^ *p* < 0.01, significantly different from AGE-BSA 200 µg/mL. Bars 400 µm.

C2C12 myotubes, a widely used model to study MW in vitro [24,25], were treated with different doses of AGE-BSA (50–800 µg/mL) (Figure 1A,B) or MGO (100–1000 µM) (Figure 1C,D) for 48 h. Both AGE-BSA and MGO induced a reduction of total myotube area starting from 100 µg/mL and 200 µM, respectively (Figure 1A,C), and a dose-dependent degradation of the most abundant sarcomeric protein, MyHC-II, with a maximum effect at 400 µg/mL and 500 µM, respectively (Figure 1B,D). Similarly, treatments with CML (400 µM) and PENT (2 µM) reduced myotube area (Figure S1A,B) and MyHC expression to a similar extent as MGO (Figure S1C). The lowest efficacious doses in reducing MyHC-II expression were then used for further investigation. Notably, MGO, PENT, and AGE-BSA induced the expression of the atrogenes *Fbxo32* and *Trim63* (Figure 1E and Figure S1D), suggesting the activation of the UPS as a common atrophy-inducing mechanism for endogenous and dietary AGEs.

Since dysregulated autophagy might contribute to muscle atrophy, we assessed whether these AGEs affected the expression of typical autophagy genes [30]. We observed that only AGE-BSA increased the expression of *Gabarap1* and *Atg12*, while both AGE-BSA and MGO upregulated the *Bnip3* gene (Figure 1F).

### 3.2. Dietary AGEs and AGE-BSA Differently Affect Mitochondrial Functions

**Figure 2:**
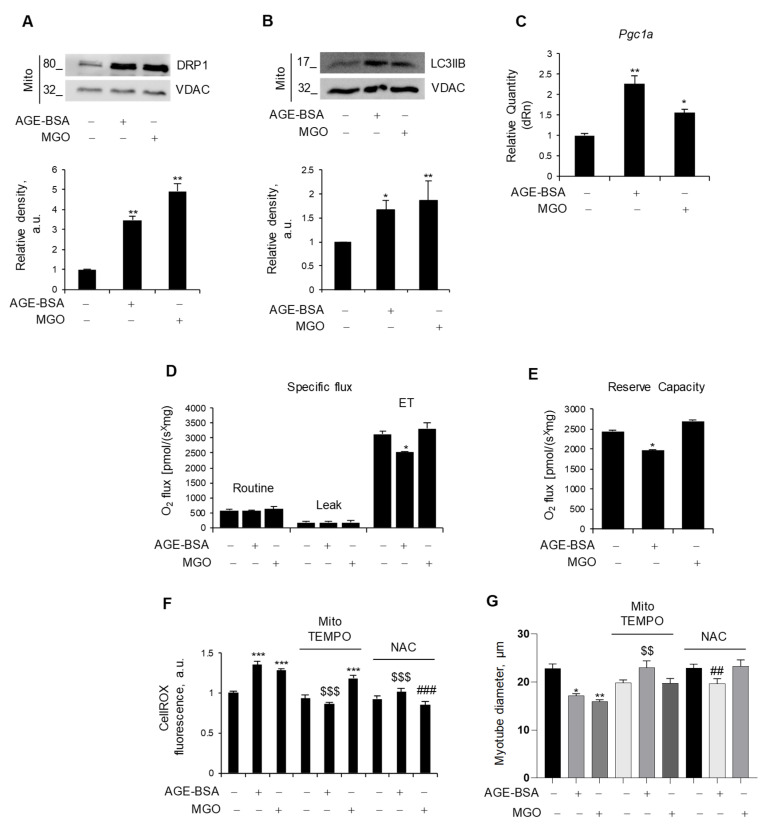
Endogenous and exogenous AGEs affect mitochondria differently. (**A**–**E**) C2C12 myotubes were treated with AGE-BSA (400 µg/mL) or MGO (500 µM) for 24 h. The fission marker, DRP1 (**A**), and the mitophagy marker, LC3IIB (**B**), were analyzed by WB in the mitochondrial fraction (Mito). Relative densities with respect to VDAC were measured. The expression of the mitochondrial biogenesis marker, *Pgc1a*, was evaluated by real-time PCR using *Gusb* as the housekeeping gene (**C**). Mitochondrial respiration, i.e., specific oxygen flux in the routine state, leakage (LEAK) state, and maximal respiratory capacity (ET) in intact myotubes, was assessed by Oroboros 2K high-resolution respirometer (**D**). Reserve respiratory capacity was reported (**E**). (**F**,**G**) C2C12 myotubes were treated with AGE-BSA or MGO with or without mitoTEMPO (10 µM) or N-acetylcysteine (NAC; 5 mM). After 24 h, ROS production was evaluated by CellROX Deep Red reagent and quantified as mean fluorescence intensity of each myotube (**F**). Myotube diameters were measured using ImageJ software (**G**). Representative images were reported (**A**,**B**). Data are means ± SEM (**G**) or SD (**A**–**F**) of three independent experiments. Statistical analysis was conducted using one-way ANOVA. * *p* < 0.05, ** *p* < 0.01, and *** *p* < 0.001, significantly different from Untr; ^$$^ *p* < 0.01 and ^$$$^ *p* < 0.001 significantly different from AGE-BSA; ^##^ *p* < 0.01 and ^###^ *p* < 0.001 significantly different from MGO.

In order to maintain metabolic efficiency and resist stress conditions, mitochondria undergo continuous remodeling through fusion and fission processes [31]. We assessed the expression levels of the fusion markers, *Opa1* and *Mfn2*, and the fission marker, *Mff*, all of which remained unchanged in myotubes in response to AGE-BSA and MGO (Figure S2). Nevertheless, both AGE-BSA and MGO were able to induce the mitochondrial accumulation of dynamin-related protein 1 (DRP1), which has a fundamental role in mitochondrial fission, and LC3IIB, a marker of mitophagy [31] (Figure 2A,B). Remarkably, both AGEs induced the expression of *Pgc1a* (Figure 2C), suggesting that mitophagy is accompanied by mitochondrial biogenesis, possibly in an attempt to replace dysfunctional mitochondria with new ones.

To evaluate the impact of AGEs on mitochondrial function, we measured oxidative respiration in intact, nonpermeabilized myotubes after 24 h of treatment. Surprisingly, only AGE-BSA impinged oxygen consumption, specifically reducing the maximal respiration capacity (ET), which was measured upon treatment with FCCP, a protonophore able to uncouple oxidation from phosphorylation, whereas MGO did not affect mitochondrial respiration (Figure 2D). In addition, AGE-BSA significantly reduced the reserve respiratory capacity (Figure 2E), a critical component of mitochondrial oxidation utilized in conditions characterized by increased ATP demand, while the oxygen consumption linked to the production of ATP was not affected.

As dysfunctional mitochondria can induce oxidative stress, C2C12 myotubes were stained with CellROX, and intracellular ROS levels were assessed following treatment with AGE-BSA or MG. Both treatments resulted in a significant increase in the fluorescence intensity, indicative of enhanced ROS production (Figure 2F). To decipher the specific source of ROS production (i.e., mitochondrial or cytosolic), two antioxidant compounds were employed during the last 24 h of AGE-BSA or MGO treatment: MitoTEMPO to scavenge selectively mitochondrial ROS or NAC to target general intracellular ROS. Co-treatment with the mitochondrial-targeted antioxidant, mitoTEMPO, prevented only the AGE-BSA-induced ROS production without altering the MGO-induced one. On the contrary, the NAC abolished the ROS production induced by both AGEs (Figure 2F). According to the concept that oxidative stress induces muscle atrophy, mitoTEMPO and NAC completely prevented the myotube diameter reduction induced by AGE-BSA and MGO, respectively (Figure 2G).

### 3.3. V. macrocarpon Extract Has Potent Anti-AGE Formation Effects

**Figure 3:**
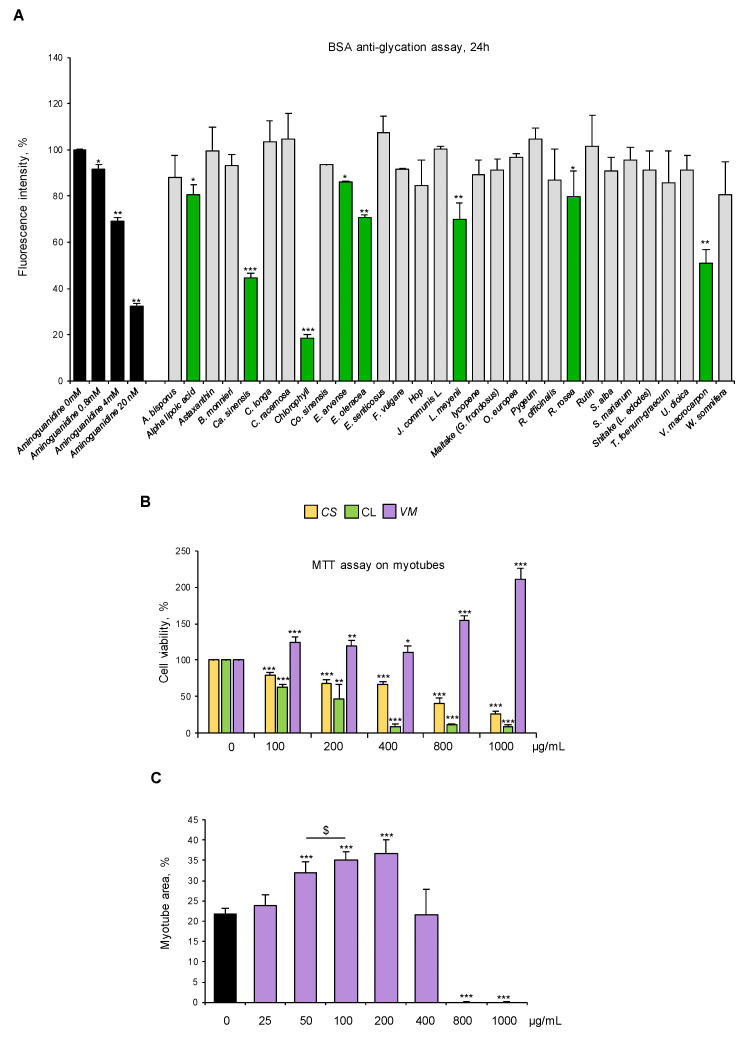
*V. macrocarpon* counteracts AGE formation without exerting any toxic effects on myotubes. (**A**) Thirty natural compounds (100 µg/mL each) were tested for their ability to counteract glyceraldehyde-derived fluorescent AGE formation at 24 h in comparison with aminoguanidine (black bars) at the indicated concentrations. Efficacious compounds were highlighted (green bars). (**B**) C2C12 myotubes were treated with different doses (0–1000 µg/mL) of *C. sinensis* (*CS*), chlorophyll (CL), or *V. macrocarpon* (*VM*), and cell viability was evaluated after 48 h by MTT. (**C**) Myotubes were treated (purple bars) or not (black bar) with *VM* (25–1000 µg/mL), and myotube areas were measured using ImageJ software after May–Grünwald/Giemsa staining (see Figure S3A for representative images). The average of myotube areas (%) is reported. Data are means ± SD (**A**,**B**) or SEM (**C**) of three independent experiments. Statistical analysis was conducted using one-way ANOVA. * *p* < 0.05, ** *p* < 0.01, and *** *p* < 0.001, significantly different from aminoguanidine (0 mM) in (**A**) or untreated myotubes (**B**,**C**); ^$^ *p* < 0.05, significantly different.

We selected standardized dry extracts from twenty-one officinal plants, four mushrooms, and five active compounds based on their proven activity as antioxidants and/or anti-inflammatory agents or their ethnobotanical use (Table 1, Table 2 and Table 3). We analyzed the ability of these natural compounds (100 µg/mL) to counteract glyceraldehyde-derived fluorescent AGE formation by using a commercial kit. Aminoguanidine was used as an anti-AGE control [32]. Eight compounds (i.e., alpha lipoic acid, *C. sinensis*, chlorophyll, *E. arvense*, *E. oleracea*, *L. meyenii*, *R. rosea*, and *V. macrocarpon*) reduced the fluorescence signal derived from AGE formation at 24 h to different extents. *V. macrocarpon*, chlorophyll (CL), and *C. sinensis* (*CS*) were particularly efficacious in inhibiting fluorescence intensity (~60%, 80%, and 50% vs. control, respectively) (Figure 3A).

Then, we tested the biological effects of *VM*, CL, and *CS* (100–1000 µg/mL) on myotube cultures. Treatment with 100 µg/mL *VM* increased cell viability by 20%, which was further increased in the presence of the highest *VM* concentrations (800–1000 µg/mL), suggesting a non-toxic effect of the extract on myotubes (Figure 3B). On the contrary, CL and *CS* dramatically reduced myotube viability starting from the lowest dose (i.e., 100 µg/mL) (Figure 3B). Thus, CL and *CS* were discarded, and *VM* underwent further investigation.

Treatment of normal C2C12 myotubes with *VM* (50–200 µg/mL) translated into an increased total myotube area (Figure 3C and Figure S3A) with a maximum effect at 100 µg/mL, without affecting myotube diameters (Figure S3B) and MyHC-II expression (Figure S3C), suggesting a positive *VM* effect on myotube growth by fusion of coexisting myoblasts in culture. To investigate the pro-myogenic potential of *VM*, we treated differentiating C2C12 myoblasts with 100 µg/mL *VM*. We found that *VM*, besides increasing myotube area (Figure S4A), increased the fusion index (FI; Figure S4B) and the number of nuclei per myotube (NpM; Figure S4C), an index of myoblast fusion, after 6 days of treatment. Moreover, *VM* induced myoblast proliferation (BrdU-positive cells) at 24 h (Figure S4D) and improved the expression of the terminal differentiation marker, embryonic MyHC, by 24 h (Figure S4E). Altogether, these results indicate that *VM* sustains myogenic differentiation and myoblast fusion into myotubes, justifying the observed increase in myotube areas (Figure 3C and Figure S3A).

### 3.4. Ultra-High Performance Liquid Chromatography–High-Resolution Mass Spectrometry (UHPLC-HRMS) Analysis of the VM Extract

To explain the effects of *VM* in inhibiting AGE formation, we analyzed the most abundant metabolites contained in the extract, which was standardized for the proanthocyanidin content. Relying upon literature data [33,34], a UHPLC-HRMS analysis was performed applying a method previously developed in our laboratory [24]. In this way, the following compounds were identified (Table 4): five anthocyanins (peonidin-3-pyranoside, malvidin-3-arabinoside, malvidin-3-pyranoside, delphidin-3-pyranoside, and petunidin-3-pyranoside); four phenolic acids (gallic acid, caffeic acid, 4-caffeoylquinic acid, and p-coumaric acid); four flavan-3-ols (procyanidin B2, catechin, procyanidin B2, and epicatechin); and three flavonols (rutin, isoquercitin, and quercitrin).

### 3.5. V. macrocarpon Counteracts AGE-BSA- and dAGE-Induced Myotube Atrophy

**Figure 4:**
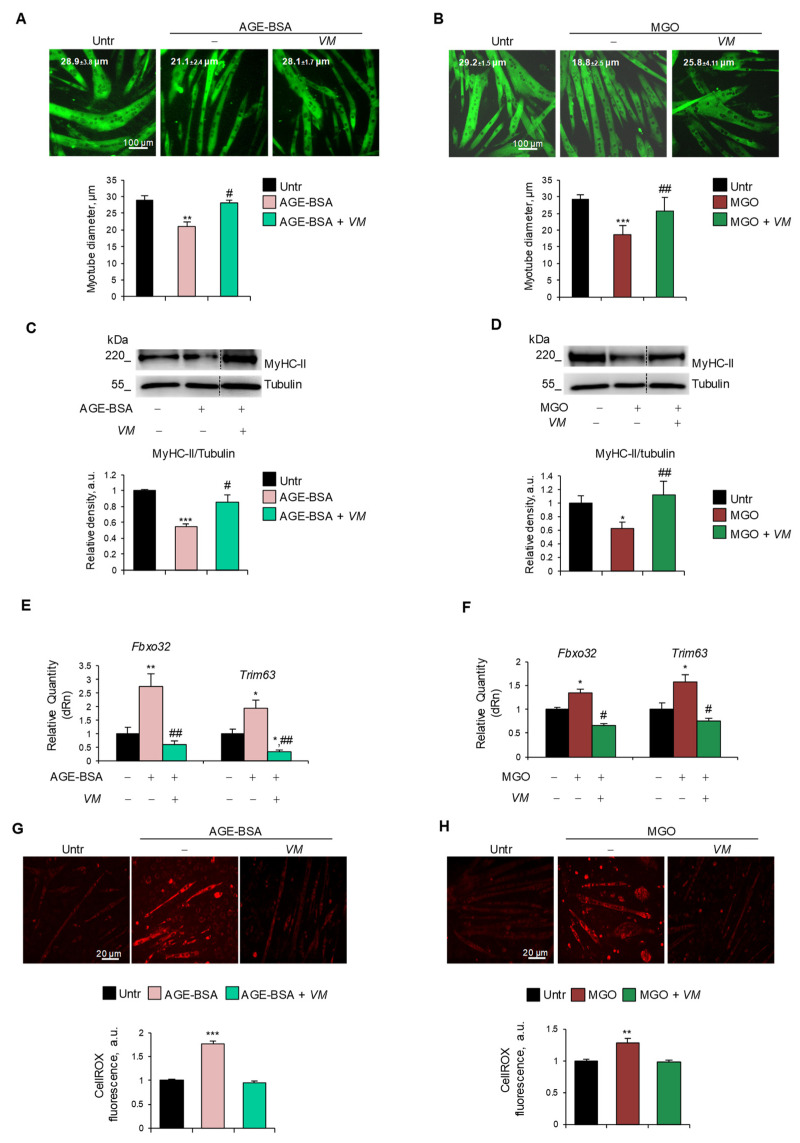
*V. macrocarpon* counteracts AGEs-induced myotube atrophy. (**A**–**H**) C2C12 myotubes were treated with AGE-BSA (400 µg/mL) or MGO (500 µM) in the absence or presence of *V. macrocarpon* (*VM*; 100 µg/mL) for 48 h (**A**–**D**) or 24 h (**E**–**H**). Immunofluorescence for MyHC-II was performed, and myotube diameters were measured using ImageJ software (**A**,**B**). MyHC expression was analyzed by Western blotting (WB). Relative densities with respect to tubulin were measured (**C**,**D**). The expression of atrogenes *Fbxo32* (Atrogin-1) and *Trim63* (MuRF1) was assessed by real-time PCR. Gene expressions were normalized on *Gapdh* (**E**,**F**). ROS production was evaluated by CellROX Deep Red reagent and quantified as the fluorescence mean intensity of each myotube (**G**,**H**). Reported are representative images (**A**–**D**,**G**,**H**) and the average of myotube diameters (**A**,**B**). Scale bar, 100 µm (**A**,**B**) and 20 µm (**G**,**H**). Data are means ± SEM (**A**,**B**,**G**,**H**) or SD (**C**–**F**) of three independent experiments. Statistical analysis was conducted using one-way ANOVA. * *p* < 0.05, ** *p* < 0.01, and *** *p* < 0.001, significantly different from untreated (Untr). ^#^ *p* < 0.05 and ^##^ *p* < 0.01 significantly different from AGE-BSA or MGO.

To assess whether *VM* was able to counteract AGE-induced atrophy, we treated C2C12 myotubes with AGE-BSA, MGO, CML, or PENT for 48 h in the absence or presence of 100 µg/mL *VM*. *VM* treatment completely abolished the reduction of myotube diameters induced by all the AGEs tested (Figure 4A,B, and Figure S5A,B). Moreover, in the presence of *VM*, AGE-BSA or MGO did not induce a decrease in MyHC-II levels (Figure 4C,D) compared to the untreated controls. Coherently, *VM* treatment blocked the AGE-dependent induction of *Fbxo32* and *Trim63* atrogenes (Figure 4E,F). In addition, *VM* inhibited the increase in ROS levels induced by both AGEs (Figure 4G,H).

In AGE-BSA- or MGO-treated myotubes, the FI (~10.5% and ~13.5%, respectively) and the NpM (~5.5 and ~6.6, respectively) were significantly lower than in untreated myotubes (~19% FI and ~10 NpM), and *VM* significantly preserved these parameters (Figure S6A,B), suggesting that *VM* might counteract the AGEs’ atrophying effects, also sustaining the myogenic potential of non-fused myoblasts and myotube growth, in accordance with the data of Figure S4.

### 3.6. AGE-BSA and MGO Induce Myotube Atrophy Through Specific Molecular Mechanisms Counteracted by V. macrocarpon

**Figure 5:**
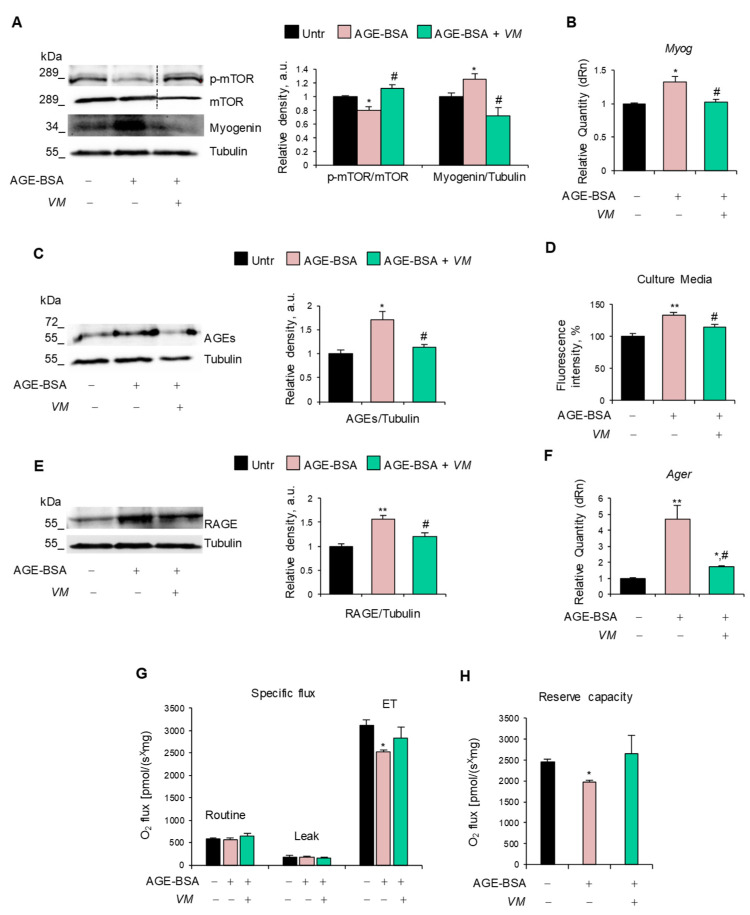
*V. macrocarpon* neutralizes the atrophying mechanisms of AGE-BSA. (**A**–**H**) C2C12 myotubes were treated with 400 µg/mL AGE-BSA (400 µg/mL) in the absence or presence of *V. macrocarpon* (*VM*; 100 µg/mL) for 48 h (**A**–**D**) or 24 h (**E**–**G**). Myogenin and phosphorylated mTOR levels were analyzed by WB. Relative densities with respect to tubulin or total mTOR were measured (**A**). The levels of the myogenin gene (*Myog*) were evaluated by real-time PCR (**B**). AGE levels were determined in cell lysates by Western blotting (WB) (**C**) and in cell culture medium (**D**) by fluorescent intensity measured at 440 nm emission and 365 nm excitation. RAGE expression was determined by WB (**E**) or real-time PCR (**F**). Mitochondrial respiration (specific oxygen flux in the routine state, leakage [LEAK] state, and maximal respiratory capacity [ET]) in intact C2C12 myotubes was assessed using an Oroboros 2K high-resolution respirometer through a substrate, uncoupler, inhibitor, titration (SUIT) protocol (**G**). Reserve respiratory capacity (**H**). Reported are representative images (**A**,**C**,**E**). Data are means ± SD. Statistical analysis was conducted using one-way ANOVA of three independent experiments. * *p* < 0.05 and ** *p* < 0.01, significantly different from untreated (Untr). ^#^ *p* < 0.05 significantly different from AGE-BSA.

The inhibitory effect of *VM* on the AGE-induced upregulation of atrogenes (Figure 4E,F) prompted us to investigate the specific pathways affected by AGE-BSA and MGO, leading to the activation of the UPS. We found that AGE-BSA decreased the activation state of anabolic mTOR and increased myogenin expression at mRNA and protein levels (Figure 5A,B), two mechanisms known to lead to UPS activation [4,35]. Notably, AGE-BSA induced further AGE accumulation in myotubes (Figure 5C) and culture media (Figure 5D) and RAGE upregulation at mRNA and protein levels (Figure 5E,F), as occurs in other atrophying conditions [36]. This suggested that RAGE signaling could mediate the AGE-BSA-dependent myotube atrophy.

*VM* interfered with all these atrophy-inducing mechanisms by restoring the activation state of mTOR, maintaining AGE, RAGE, and myogenin expressions at similar levels to untreated myotubes, and preserving the maximal respiration and reserve respiratory capacity altered by AGE-BSA (Figure 5G,H).

**Figure 6:**
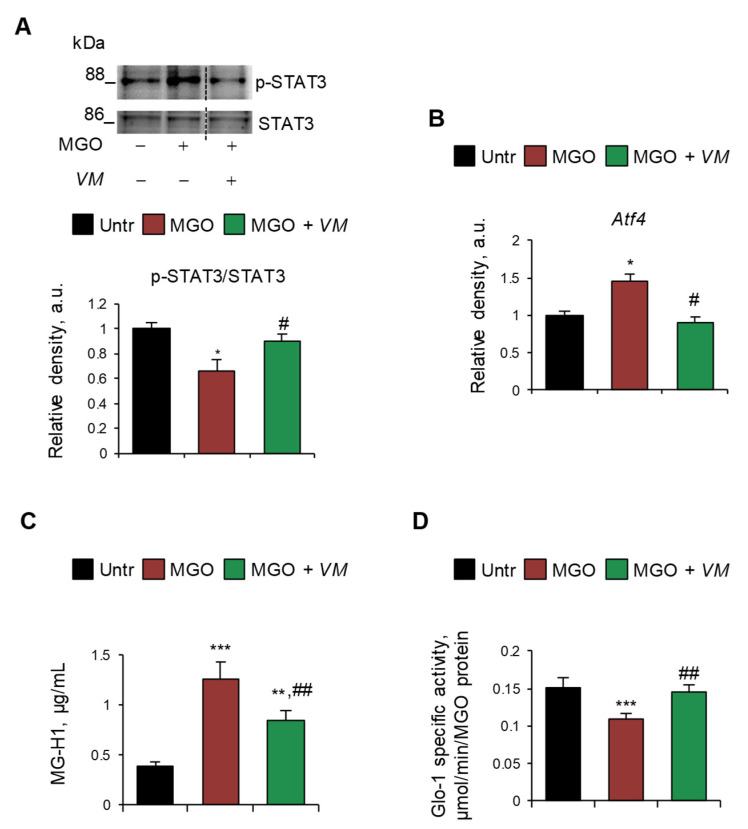
MGO-dependent glycative stress is blunted by *V. macrocarpon* in protecting myotubes from atrophy. (**A**–**D**) C2C12 myotubes were treated with MGO (500 µM) in the absence or presence of *V. macrocarpon* (*VM*; 100 µg/mL). After 6 h, phosphorylated (i.e., activated) STAT3 levels were analyzed by WB. Relative densities with respect to total STAT3 were reported (**A**). The expression of the *Atf4* was evaluated after 1 h of treatment by real-time PCR using *Gusb* as the housekeeping gene (**B**). After 24 h, the amounts (µg/mL) of MG-H1 were quantified by ELISA (**C**). Glo-1 specific activity was measured in the culture medium (**D**). Reported are representative images (**A**). Data are means ± SD. Statistical analysis was conducted using one-way ANOVA of three independent experiments. * *p* < 0.05, ** *p* < 0.01, and *** *p* < 0.001, significantly different from untreated (Untr). ^#^ *p* < 0.05 and ^##^ *p* < 0.01 significantly different from MGO.

On the contrary, we did not observe any upregulation of RAGE (mRNA and protein) and myogenin or deactivation of mTOR upon treatment with MGO or other dAGEs (Figure S7A,B), suggesting a RAGE-independent mechanism underlying the atrophying effects of exogenous AGEs. Thus, we investigated alternative pathways, including the involvement of glycative stress in MGO-induced myotube atrophy. We found that MGO activated the catabolic STAT3, which was unaffected by AGE-BSA (Figure S7C), and induced the expression of *Atf4* (Figure 6A,B), which is known to promote muscle atrophy [37]. Moreover, MGO induced the specific accumulation of MGO-derived hydroimidazolone (MG-H1) in myotubes, as evaluated by ELISA dosage (Figure 6C), and simultaneously reduced the enzyme-specific activity of glyoxalase 1 (Glo1) (Figure 6D), the major enzyme involved in the removal of MGO, suggesting the occurrence of a glycative stress condition [38]. Interestingly, *VM* prevented the MGO-dependent upregulation of STAT3 and *Atf4* and partially rescued MG-H1 intracellular levels (Figure 6A–C). Finally, *VM* completely restored Glo1 specific enzyme activity in the presence of MGO (Figure 6D).

## 4. Discussion

AGEs are harmful compounds formed especially when proteins combine with sugars in an enzyme-independent process called glycation. In addition to endogenous AGEs (typically, serum glycated albumin) produced naturally in the body during metabolism, especially in hyperglycemic conditions and during aging, exogenous final or precursor AGEs can be introduced with prepackaged or ultra-processed foods, refined carbohydrates, red meat, high-sugar drinks, and foods typical of WD [8]. Noteworthy, WD consumption strongly impacts metabolism, inflammation, and antioxidant status, mainly due to the interaction of AGEs with their primary receptor, RAGE, thus predisposing to many non-communicable diseases (e.g., obesity, diabetes, cardiovascular disease, cancers, and sarcopenia, i.e., the loss of muscle mass and performance occurring during aging) [8,9,39]. The effects exerted and the mechanisms used by endogenous AGEs to induce muscle atrophy have been demonstrated in several in vitro and in vivo studies, especially using AGE-BSA. Indeed, endogenous AGEs act through RAGE to achieve the following: (i) reduce myogenesis [17]; (ii) disrupt muscle protein balance by interfering with protein synthesis through the Akt/mTOR pathway and by accelerating protein breakdown through AMP-activated protein kinase (AMPK)-dependent UPS and ALS activation [17]; (iii) induce oxidative stress, mitochondrial dysfunction, and energy deficiency leading to reduced ATP production [40]; and (iv) impair insulin signaling by upregulating STAT3 and downregulating ERK activities, preventing muscles from absorbing nutrients for growth and repair [36].

Although the contribution of dAGEs to skeletal muscle dysfunction in humans had been hypothesized by several authors [12], the direct impact of exogenous AGEs in inducing muscle atrophy deserved elucidation. Here, we investigated the direct effects of different exogenous AGEs on skeletal muscle homeostasis in vitro in comparison with AGE-BSA. We found that the exogenous final AGEs tested (i.e., CML and PENT) and the highly reactive MGO, which are characterized by different formation processes, thus mimicking the intake of WD processed foods [10,27,28], were able to induce a ~30% reduction in myotube size and MyHC degradation at specific doses.

We found common molecular mechanisms, i.e., accumulation of ROS, activation of the UPS, and mitochondrial damage, underpinning the atrophying effects of both AGE-BSA and MGO. In addition, and in line with published results obtained in myoblasts [17], our data demonstrated an anti-myogenic effect of both AGE-BSA and MG, indicating that the inhibition of differentiation or fusion of mononucleated myoblasts into myotubes might potentially contribute to AGE-induced atrophy.

Although the involvement of ROS production as a mechanism used by AGEs to induce muscle atrophy is known [40], our results point to ROS production as the primary cause of AGE-induced atrophy. Indeed, the results obtained using the mitochondrion-specific antioxidant, mitoTEMPO, and the pan-antioxidant NAC demonstrated that mitochondrial ROS or ROS from cellular sources other than mitochondria have a major role in inducing myotube diameter reduction by AGE-BSA or MGO, respectively. Notably, independent of its origin, oxidative stress appears to be the key factor triggering muscle atrophy in the presence of both AGEs. Thus, the activation of UPS might be a secondary event in AGE-dependent atrophy, considering the ability of ROS to directly activate various catabolic signaling pathways leading to atrogene upregulation [5]. CML did not upregulate atrogenes, in accordance with recent data demonstrating insulin resistance and upregulation of pro-inflammatory cytokines as mechanisms used by CML to induce myotube atrophy [41].

The different origins of ROS induced by AGE-BSA and MGO might also explain the modulation of specific signaling pathways in atrophic myotubes. Indeed, AGE-BSA reduced the anabolic mTOR, and MGO activated the catabolic STAT3 transcription factor, suggesting that AGE-BSA might reduce MyHC-II expression not only by upregulating atrogenes but also by decreasing protein synthesis. Accordingly, elevated ROS levels suppress the PI3K/AKT/mTOR pathway and consequently inhibit protein synthesis in atrophying conditions [42], and MGO induces extramitochondrial ROS production in myoblasts by STAT3 [43], culminating in UPS activation and excessive MyHC degradation. In line with MGO treatment, we observed upregulated *Atf4*, a transcription factor able to induce atrogenes expression [37]. Even though only MGO induced the expression of the autophagic markers *Gabarap* and *Atg12*, both AGEs upregulated the expression of *Bnip3*, suggesting the presence of dysfunctional mitochondria to be eliminated through mitophagy. Although mRNA levels of autophagy-related genes are not sufficient to infer autophagic flux or autophagolysosome formation, the increased expressions of *Atg12, Gabarap*, and *Bnip3* suggest activation of autophagic and mitophagic programs in response to AGE treatment. Previous studies on C2C12 myotubes have shown that starvation-induced autophagy can be predominantly regulated at the post-transcriptional level, with increased protein expression arising from translation of preexisting mRNA pools [44]. In this context, the increased LC3IIB content in mitochondrial fractions, especially in AGE-BSA-treated cells, observed in our study, represents a more direct indicator of mitophagy activation. In contrast, conclusions regarding bulk autophagy remain tentative, given the absence of protein-level analyses and the lack of autophagic flux assessment, which are necessary to distinguish between increased autophagosome formation and impaired degradation. Future studies should aim to integrate dynamic assessments of autophagy to better characterize the contribution of transcriptional versus post-transcriptional mechanisms in AGE-induced atrophy conditions.

Regardless of the lack of effect on the expression of genes involved in mitochondrial dynamics, the accumulation of DRP1 in mitochondria of AGE-BSA- and MG-treated cells suggests that mitochondria undergo fission, an event that usually precedes mitophagy [45]. We can speculate that AGE-damaged mitochondria are constantly replaced, as suggested by the induction by both AGEs of PGC-1α, a marker of mitochondrial biosynthesis [46]. Notably, only AGE-BSA, which induced an increase in mitochondrial ROS, affected O_2_ consumption in non-permeabilized myotubes, reducing the maximal respiration capacity and reserve respiratory capacity, a critical component of mitochondrial oxidation that can be utilized during states of increased energy demand.

However, the most intriguing difference in the molecular mechanisms activated by endogenous and exogenous AGEs in inducing myotube atrophy was the involvement of further AGE accumulation and the role of RAGE. AGE-BSA, in accordance with previous results [36], induced further intracellular AGE accumulation in myotubes and in culture media, suggesting the presence of a vicious circle in which mitochondrion-derived ROS might lead to AGE formation and further oxidative stress. Moreover, as in the case of the RAGE ligand S100B [35], RAGE was upregulated in myotubes upon AGE-BSA treatment at protein and mRNA levels, concomitantly with increased myogenin expression and MyHC-II degradation, suggesting that RAGE signaling might mediate AGE-BSA-dependent atrophying effects. Instead, MGO, CML, and PENT did not affect RAGE expression, suggesting that dAGEs might act in a RAGE-independent manner. RAGE knockdown or knockout experiments in myotubes exposed to dAGEs will demonstrate the effective role of RAGE in the dAGE’s atrophying mechanisms. However, it is known that MGO and its final AGE, MG-H1, which was overproduced in MGO-treated myotubes, exert significant biological effects, such as inflammation and toxicity, through RAGE-independent mechanisms [47]. The accumulation of MG-H1 in the presence of MG was concomitant with the reduced activity of Glo-1, indicating the inability of the enzyme to detoxify MG and the induction of glycation stress.

Collectively, our data demonstrated the dangerous direct effects of several exogenous AGEs in myotubes and elucidated the molecular mechanisms used by AGE-BSA to induce atrophy.

Some chemically synthesized drugs effectively inhibit the formation/activity of AGEs via different mechanisms. For example, aminoguanidine inhibits the formation of AGEs by trapping active dicarbonyl compounds, whereas the chemical inhibitor ALT removes the cross-linked products, and metformin reduces glycemia. However, these compounds cause severe side effects in humans, limiting their long-term clinical applications [22]. At present, natural compounds with antioxidant properties, including polyphenols, polysaccharides, terpenoids, vitamins, alkaloids, and peptides [22], are the most promising research direction in inhibiting AGE formation, displaying good activity and safety. Following this road, we found that starting from thirty natural compounds, *VM*, *C. sinensis*, and chlorophyll showed a surprising ability to counteract AGE formation in vitro. However, when tested for their biological properties, only *VM* (100 µg/mL) was not toxic and able to sustain the viability of muscle precursor cells.

*VM*, commonly known as American cranberry, is a native fruit from North America containing low carbohydrate concentrations in comparison with other fruits and a high content of vitamins (especially vitamin C), polyphenolic and flavonoid compounds, and minerals. Thus, cranberry has a wide variety of biological activities, including antibacterial, anticarcinogenic, antiangiogenic, anti-inflammatory, and antioxidant effects [48].

We found that the addition of *VM* did not translate into a hypertrophic effect, evaluated as myotube diameters and MyHC expression, in physiological conditions; whereas, it stimulated myoblast proliferation, terminal differentiation, and fusion into myotubes. *VM* maintained the myogenic potential of non-fused myoblasts also in the presence of AGEs, ensuring myotube trophism.

Importantly, *VM* counteracted the reduction in myotube diameter and MyHC-II expression induced by both endogenous and dietary AGEs, thereby restraining their atrophying effects. Indeed, in the presence of *VM*, neither AGE-BSA nor MG activated their atrophying mechanisms, including increased ROS production and mitophagy and activation of the UPS. Moreover, *VM* maintained the activation state of mTOR and STAT3 at levels similar to those in untreated controls in the presence of AGE-BSA or MGO, respectively. Notably, *VM* counteracted the AGE-BSA-dependent upregulation of the RAGE-myogenin axis and reduced intracellular and extracellular accumulation of AGEs in the same condition. Similarly, *VM* abolished the MGO-dependent accumulation of MG-H1 by reducing the glycation stress and *Atf4* upregulation.

Besides proanthocyanidins (PACs) [49,50], which were used to standardize our *VM* extract and have proven anti-glycation properties, mass spectrometry analysis revealed that *VM* extract contains a cocktail of secondary metabolites known to limit AGE formation/accumulation. Indeed, resveratrol is a strong glycation inhibitor that reduces reactive sugar levels and MGO conjugation, and it can also inhibit AGE receptors [51,52]; quercetin is considered a finer substitute to aminoguanidine for its ability to inhibit early, intermediate, and late stages of glycation products [53]; myricitrin and its derivatives have a strong antiglycation activity by spontaneous interactions with BSA, impairining the cross-linking with reducing sugars [54]. Among polyphenols, the presence of caffeic acid, rutin, catechin, and anthocyanins such as malvidin justifies the inhibitory activity of *VM* on AGE formation and resistance to AGE-dependent pathogenic effects [55,56,57]. In particular, anthocyanin-3-glucoside inhibits nonenzymatic glycation by stabilizing through direct spontaneous binding the α-helical structure of the proteins [58]. Additionally, some polyphenol compounds, such as gallic acid and curcumin, attenuate the expression of RAGE, thus justifying the protective effect of *VM* from RAGE-dependent effects in the presence of AGE-BSA [59]. Finally, the diverse vitamins (vitamin B1, B3, D, and E) and minerals (Mo, Mn, and Mg) contained in *VM* [60] show significant glycation-inhibitory properties. Based on the strong ability of *VM* to reduce AGE formation/accumulation in vitro, further studies should evaluate the pharmacokinetics of its active compounds, including the extent of absorption. PACs, which are considered the main responsible for *VM*’s activity in several in vivo conditions [61], are characterized by a low (<10%) absorption. However, clinical trials demonstrated that even small amounts of *VM* compounds absorbed through the gastrointestinal tract can exert systemic effects, with their bioavailability increasing in long-term *VM* administration [62,63,64]. Moreover, it should be taken into account that hepatocytes, enterocytes, and the gut microbiota can metabolize cranberry compounds, converting them into biologically active metabolites (e.g., PACs’ and flavonoids’ derivatives), as identified in human plasma and urine [65].

## 5. Conclusions

Our research focused on the atrophying effects of AGEs on muscle, and encouragingly, *VM* appears to prevent the accumulation/activity of both WD-derived and endogenous AGEs at the muscle level, offering a promising dietary intervention for mitigating AGE-induced muscle atrophy. Considering previous results showing that high levels of serum and muscle AGEs were associated with loss of physical performance and muscle atrophy in diabetic patients and sarcopenic subjects, *VM* consumption might potentially delay the loss of muscle mass in several atrophying conditions, including aging. At the same time, our data open future research directions to investigate the potential beneficial effects of *VM* in combating AGEs’ adverse effects by promoting several pathologies. Although the most traditional use of *VM* is to prevent urinary tract infections [47], emerging observational and interventional studies in humans indicate that the consumption of cranberry or cranberry-derived products might be useful in counteracting the metabolic syndrome, a complex condition characterized by multiple cardiovascular risk factors, including obesity, hypertension, and hyperglycemia [47], in which AGEs have been extensively implicated [9,39]. Further investigations in preclinical models and clinical trials could consolidate our findings to verify the efficacy of *VM* extract in reducing AGE accumulation/activity in tissues in order to validate *VM* supplementation as a prevention tool for WD-dependent muscle atrophy, also considering the non-toxic effects reported for this herbal extract [64].

## Figures and Tables

**Table 1:** Plants tested in this study, including their vernacular and botanical names, organ(s) used, origin, titrated active compounds, solvent for extraction, and drug and extract ratio (DER).

Standardized Dried Extracts of Officinal Plants						
Vernacular Name	Scientific Name	Organ Used	Origin	Titrated Active Compounds	Solvent	DER
**Water hyssop**	*Bacopa monnieri* (L.) Wettst.	Aerial Part	India	20% bacosides	Ethanol	12–20:1
**Tea**	*Camellia sinensis* (L) Kuntze	Leaf	China	50% polyphenols	70% water 30% ethanol	5–10:1
**Turmeric**	*Curcuma longa* L.	Root	India	25% curcuminoids	30% water 70% ethanol	5–10:1
**Black cohosh**	*Cimicifuga racemosa* (L.) Nutt	Root		3% L-triterpene glycosides	60% water 40% ethanol	8–10:1
**Common** **Horsetail**	*Equisetum arvense* L.	Aerial Part	Italy	10% silica	Water	5–10:1
**Acai**	*Euterpe oleracea* Mart.	Fruit	Brasil	10% polyphenols	20% water 80% ethanol	5–10:1
**Siberian Ginseng**	*Eleutherococcus senticosus* Maxim	Radix	Russia	20% ginsenosides	30% water 70% ethanol	4–10:1
**Fennel**	*Foeniculum vulgare* Mill	Fruit	Egypt	0.5% essential oil	30% water 70% ethanol	5–10:1
**Hop**	*Humulus lupulus* L.	Strobilus	Serbia	0.4–0.5% rutoside	80% water 20 ethanol	4:1
**Common Juniper**	*Juniperus communis* L.	Seed	Macedonia		water	4:1
**Maca**	*Lepidium meyenii* Walp.	Root	Perù	1% glucosinolates	30% water 70% ethanol	15:1
**Olive**	*Olea europaea* L.	Leaf	Morocco	6% oleuropein	30% water 70% ethanol	5:1
**African cherry**	*Pygeum africanum* Hook. f.	Bark	Africa		20% water 80% ethanol	5–10:1
**Rosemary**	*Rosmarinus officinalis* L.	Leaf	Albania	10% terpenes	70% water 30% ethanol	4–10:1
**Arctic root**	*Rhodiola rosea* L.	Root	Russia	3% rosavin	40% water 60% ethanol	5:1
**White willow**	*Salix alba* L.	Bark	Albania	15% salicin	30% water 70% ethanol	12–15:1
**Milk thistle**	*Silybum marianum* (L.) Gaertn.	Fruit	Austria		30% water 70% ethanol	4:1
**Fenugreek**	*Trigonella foenum-graecum* L.	Seed	India	80% silymarin	20% water 80% ethanol	30:1
**Stinging nettle**	*Urtica dioica* L.	Leaf	Bulgaria	0.8% betasitosterol	30% water 70% ethanol	4:1
**Cranberry**	*Vaccinium macrocarpon* Aiton	Fruit	Ukraine	40% proanthocyanidins	30% water 70% ethanol	20–30:1
**Ashwagandha**	*Withania somnifera* (L.) Dunal	Root	India	2% withanolides	20% water 80% ethanol	3/6:1

**Table 2:** Mushrooms tested in this study, including their vernacular and botanical names, organ(s) used, origin, titrated active compounds, and solvent for extraction.

Mushrooms					
Vernacular Name	Scientific Name	Organ Used	Origin	Trited Active Compounds	Solvent
**Almond mushroom**	*Agaricus blaxei* Murrill	sporophorum	China	10% polysaccarides	30% water 70% ethanol
**Caterpillar fungus**	*Cordyceps sinensis* (Berk.) Sacc.	sporophorum	China	40% polysaccarides	40% water 60% ethanol
**Maitake**	*Grifola frondosa* (Dicks.) Gray	sporophorum	China	20% polysaccarides	30% water 70% ethanol
**Shitake**	*Lentinula edodes* (Berk.) Pegler	sporophorum	China	10% polysaccarides	30% water 70% ethanol

**Table 3:** Active compounds tested in this study.

Active Compounds		
Name	Origin	Assay
**Alpha lipoic acid**	synthetic	≥98%
**Astaxantin**	from *Haematococcus pluvialis*	≥5%
**Chlorophyllin**	from *Heliantus annuus* seed	≥18%
**Lycopen**	from *Solanum lycopersicum*	5% Lycopene
**Rutin**	from *Sophora japonica*	95%

**Table 4:** Results of LC-MS analysis. Compounds are listed according to their retention time (Rt), recorded with the applied method. Possible sugar moieties are specified for anthocyanins (^a^). Phenol was identified based on the formation of a radical cation (M^+^.) in the positive ion mode (^b^). Only the fragment with the highest intensity is reported. Missing product ions are due to low signal abundance (^c^).

Label	Compound	Sugar Moiety ^a^	Formula	Mass	Rt (min)	Precursor Ion (*m*/*z*)	Product Ion (*m*/*z*) ^c^	Polarity
1	Gallic acid		C_7_H_6_O_5_	170.0215	1.7	169		Negative
2	Caffeic acid		C_9_H_8_O_4_	180.0400	3.1	179		Negative
3	Procyanidin B2		C_30_H_26_O_12_	578.1421	3.9	577	289	Negative
4	Rutin		C_27_H_30_O_16_	610.1533	4.0	609		Negative
5	4-Caffeoylquinic acid		C_16_H_18_O_9_	354.0900	4.2	353	191	Negative
6	p-Coumaric acid		C_9_H_8_O_3_	164.0400	4.2	163		Negative
7	Peonidin-3-pyranoside	Glucose/ galactose	C_22_H_23_O_11_	463.1240	4.3	463 M^+ b^		Positive
8	Malvidin-3-arabinoside		C_22_H_23_O_11_	463.1240	4.4	463 M^+ b^	331	Positive
9	Catechin		C_15_H_14_O_6_	290.0800	4.5	289	203	Negative
10	Malvidin-3-pyranoside	Glucose/ galactose	C_23_H_25_O_12_	493.1353	4.5	493 M^+ b^	331	Positive
11	Isoquercitin		C_21_H_20_O_12_	464.0973	4.9	463	300	Negative
12	Delphidin-3-pyranoside	Glucose/ galactose	C_21_H_21_O_12_	465.1027	5	465 M^+ b^	303	Positive
13	Petunidin-3-pyranoside	Glucose/ galactose	C_22_H_23_O_12_	479.1183	5.1	479 M^+ b^	301	Positive
14	Quercitrin		C_21_H_20_O_11_	448.1005	5.2	447	344	Negative
15	Procyanidin A2		C_30_H_24_O_12_	576.1263	5.4	575	226	Negative
16	Epicatechin		C_15_H_14_O_6_	290.0788	5.7	289	203	Negative

## Data Availability

Data is contained within the article and Supplementary Materials.

## Supplementary Materials

The following supporting information can be downloaded at: https://www.mdpi.com/article/10.3390/antiox14080900/s1. Table S1. List of primary and secondary antibodies used in WB; Table S2. List of primers used in real-time PCR; Figure S1. The dietary AGEs, CML, and PENT, induced myotube atrophy; Figure S2. Endogenous or exogenous AGEs do not affect mitochondrial dynamic markers; Figure S3. Effects of *V. macrocarpon* on normal myotubes; Figure S4. *V. macrocarpon* increases myoblast proliferation, terminal differentiation, and fusion into myotubes; Figure S5. *V. macrocarpon* counteracts CML- or PENT-induced myotube atrophy; Figure S6. Diet-derived AGEs and AGE-BSA used different atrophying molecular mechanisms; Figure S7. *V. macrocarpon* preserves myotube growth in the presence of endogenous or exogenous AGEs.

## Abbreviations

The following abbreviations are used in this manuscript:

AGEs	advanced glycation end-products
AGE-BSA	glycated albumin
ALS	autophagy-lysosomal system
Akt	protein kinase B
CL	chlorophyll
CML	carboxymethyl-L-lysine
*CS*	*Camellia sinensis*
CSA	cross-sectional area
Dex	dexamethasone
dAGEs	diet-derived AGEs
FI	fusion index
MG-H1	5-hydro-5-methylimidazolone
MGO	Methylglyoxal
mTOR	mammalian target of rapamycin
MyHC-II	type II myosin heavy chain
MW	muscle wasting
NAC	N-Acetyl-L-Cysteine
NCDs	noncommunicable chronic diseases
NpM	nuclei per myotube
PENT	pentosidine
RAGE	receptor for AGE
ROS	reactive oxygen species
UPS	ubiquitin–proteasome system
*VM*	*Vaccinium macrocarpon*
WD	Western diet
