# Supplementary material for: Diet-Derived Advanced Glycation End-Products (AGEs) Induce Muscle Wasting In Vitro, and a Standardized Vaccinium macrocarpon Extract Restrains AGE Formation and AGE-Dependent C2C12 Myotube Atrophy
Source: Antioxidants (Basel). 2025 Jul 23;14(8):900. doi: 10.3390/antiox14080900 (PMC12383208; doi:10.3390/antiox14080900)
Supplement: Supplementary file 1 [file antioxidants-14-00900-s001.zip › antioxidants-3684440-supplementary.pdf]

Supplemental Materials

Table S1. List of primary and secondary antibodies used in WB.

**Primary Antibody**	**Source**	**Molecular weight (kDa)**	**Dilution**	**Secondary Antibody** (Sigma-Aldrich)	**Dilution**
Mouse monoclonal anti-MyHC-II (MF20)	eBiosciences	220	1:10000	Goat anti-mouse IgG/IgM-HRP conjugated	1:100.000
Mouse monoclonal anti-MyHC developmental (RNMY2/9D2)	Monosan	220	1:500	Goat anti-mouse IgG/IgM-HRP conjugated	1:5000
Mouse monoclonal l anti-Myogenin (F5D)	Santa Cruz Biotech	34	1:1000	Goat anti-mouse IgG/IgM-HRP conjugated	1:1000
Mouse monoclonal anti-α-Tubulin (DM1A)	Santa Cruz Biotech	55	1:2000	Goat anti-mouse IgG/IgM-HRP conjugated	1:5000
Rabbit polyclonal anti-phospho-mTOR (Ser2448)	Cell Signaling Tech.	289	1:1000	Goat anti-rabbit IgG-HRP conjugated	1:2000
Rabbit polyclonal anti-mTOR	Cell Signaling Tech	289	1:1000	Goat anti-rabbit IgG-HRP conjugated	1:2000
Mouse monoclonal anti-β-actin	Santa Cruz Biotech	45	1:1000	Goat anti-mouse IgG/IgM-HRP conjugated	1:5000
Rabbit polyclonal anti-phospho-STAT3 (Y705)	ABclonal	88	1:1000	Goat anti-rabbit IgG-HRP conjugated	1:2000
Rabbit polyclonal anti-STAT3	ABclonal	88	1:1000	Goat anti-rabbit IgG-HRP conjugated	1:2000
Rabbit monoclonal anti-VDAC.	Cell Signaling Tech	32	1:1000	Goat anti-rabbit IgG-HRP conjugated	1:10000
Rabbit polyclonal anti-LC3B	Proteintech	15	1:1000	Goat anti-rabbit IgG-HRP conjugated	1:10000
Mouse monoclonal anti-DLP1 (8/DLP)	BD Transduction Laboratories	79-84	1:1000	Goat anti-mouse IgG/IgM-HRP conjugated	1:10000
Mouse monoclonal anti-AGE	Cosmo Bio Co.,LTD	43-75	1:1000	Goat anti-mouse IgG/IgM-HRP conjugated	1:2000
Goat polyclonal anti-RAGE (N-16)	Santa Cruz Biotech	55	1:1000	Rabbit anti-goat IgG-HRP conjugated	1:2000

Table S2. List of primers used in real-time PCR.

**Gene**	**Forward primer 5'-3'**	**Reverse primer 5'-3'**	**Gene Accession Number**
Fbxo32	GTCTTGGGAATGTACGACGC	GCGCTCCTTCGTACTTCCTT	NM_026346.3
Trim63	ATTGTAGAAGCCTCCAAGGG	GGTGTTCTTCTTTACCCTCTGTG	NM_001039048.2
Gapdh	GCCTTCCGTGTTCCTACCC	CAGTGGGCCCTCAGATGC	NM_001411845.1
Ager	CACTTGTGCTAAGCTGTAAGGG	CATCGACAATTCCAGTGGCTG	XM_006523501.4
Myogenin	CTGGGGACCCCTGAGCATTG	ATCGCGCTCCTCCTGGTTGA	NM_031189.2

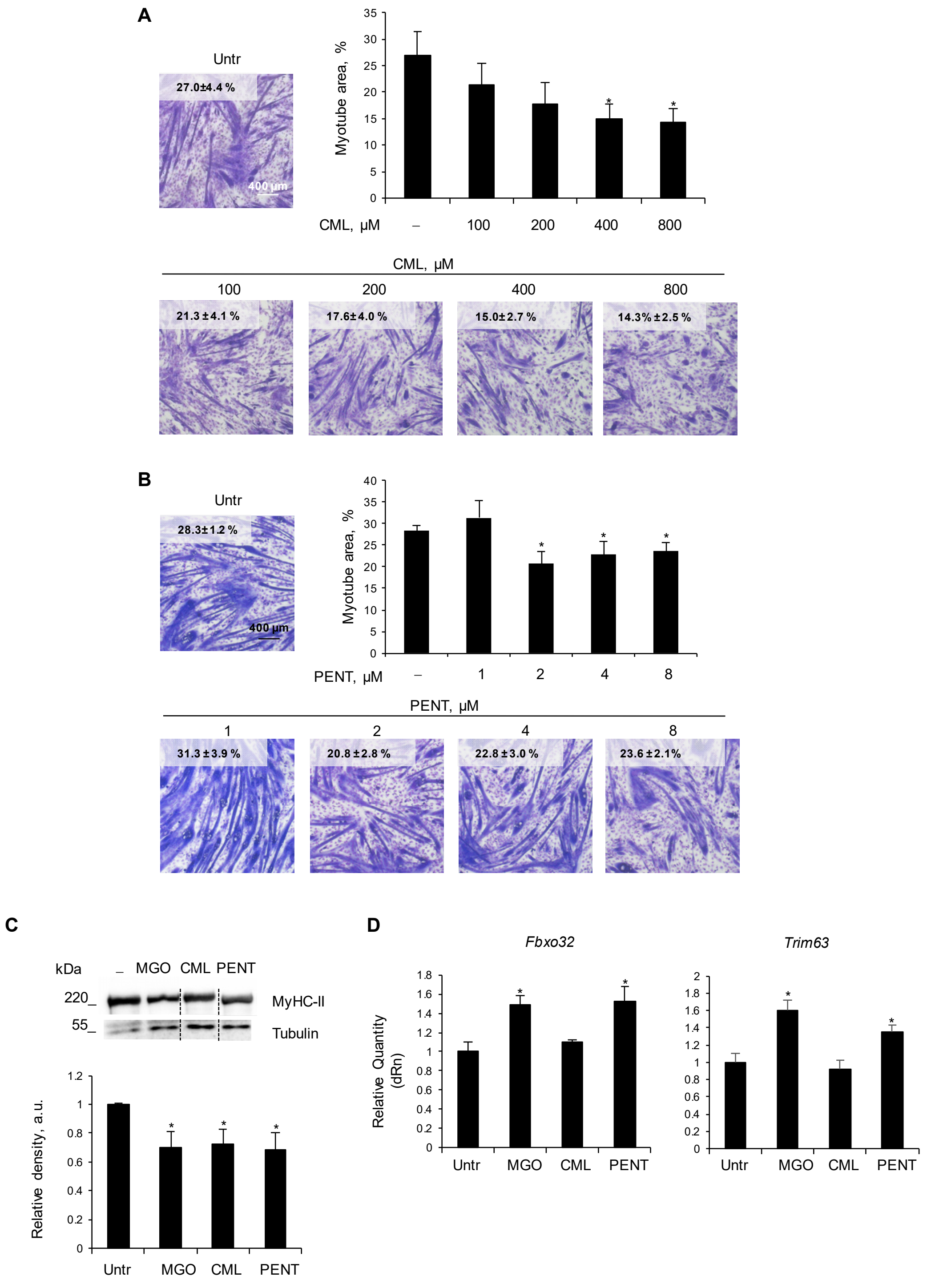

Figure S1. The dietary AGEs, CML and PENT, induced myotube atrophy. (A-D) C2C12 myotubes obtained by culturing myoblasts in differentiation medium (DM) for 4 days were treated with different doses of the dietary CML, pentosidine (PENT) or methylglyoxal (MGO) (500 µM) for 48 h (A-C) or 24 h (D). May-Grünwald/Giemsa staining was performed and myotube areas were measured using *Image J* software. The average of myotube area (%) is reported (A,B). WB analysis for MyHC-II was performed with tubulin used as loading control. Relative densities were measured with respect to tubulin (C). Levels of *Fbxo32* and *Trim63* were assessed by real-time PCR using *Gapdh* as the housekeeping gene (D). Reported are representative images (A-C). Data are means ± SEM (A,B) or SD (C,D) of three independent experiments. Statistical analysis was conducted using one-way ANOVA. *p<0.05, significantly different from Un

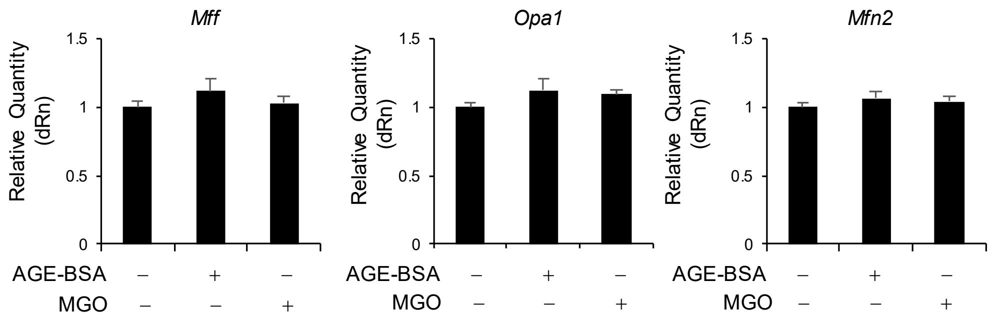

Figure S2. Endogenous or exogenous AGEs do not affect mitochondrial dynamic markers. C2C12 myotubes obtained by culturing myoblasts in differentiation medium (DM) for 4 days were treated with AGE-BSA (400 µg/mL) or methylglyoxal (MGO; 500 µM) for 24 h. The expression of *Mff* (fission), and *Opa1* and *Mfn2* (fusion) was assessed by real-time PCR using *Gusb* as the housekeeping gene. Data are means ± SD of three independent experiments. Statistical analysis was conducted using one-way ANOVA.

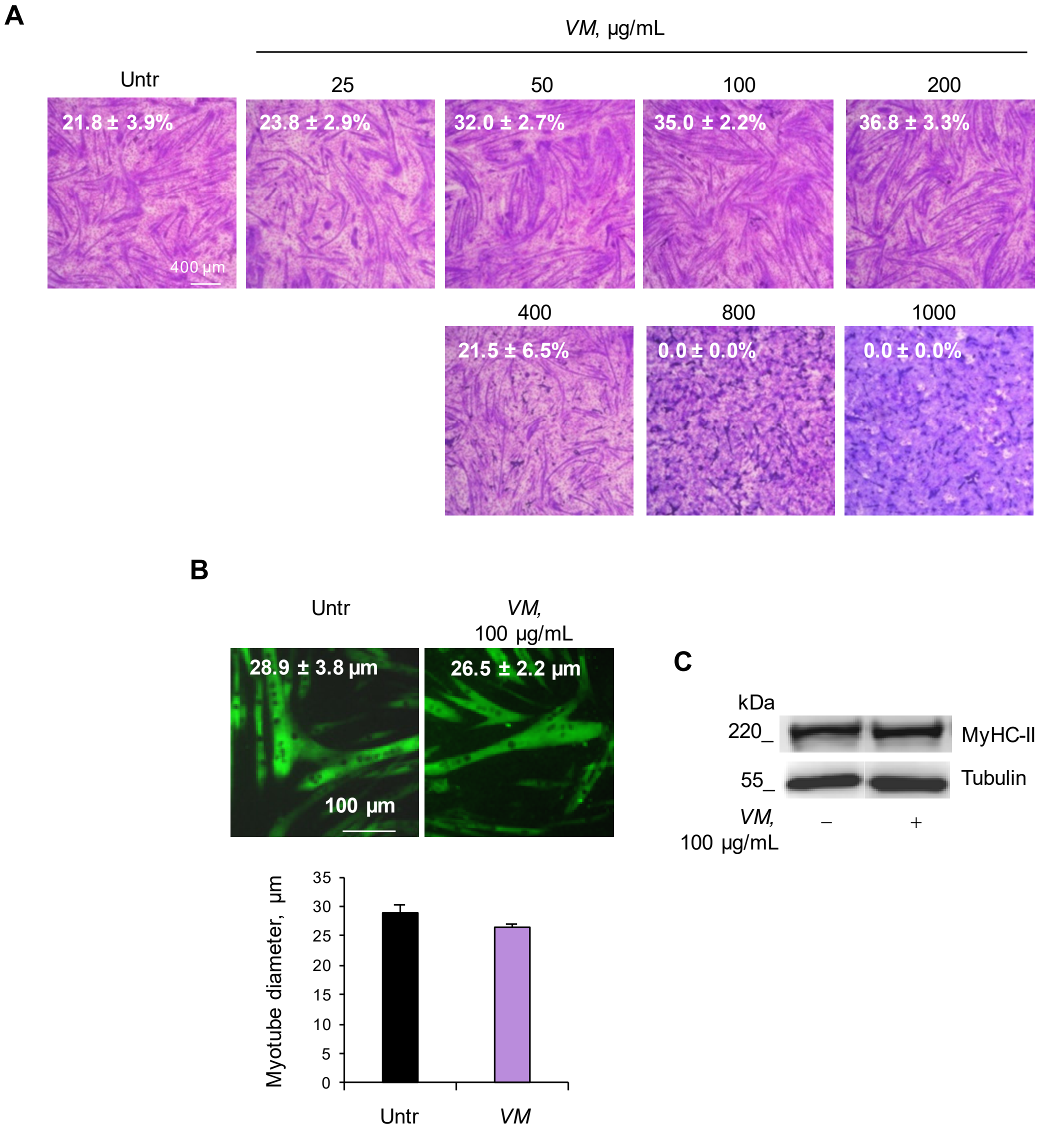

Figure S3. Effects of *V. macrocarpon* on normal myotubes. (A-C) C2C12 myotubes obtained by culturing myoblasts in differentiation medium (DM) for 4 days, were treated with different doses of *V. macrocarpon* (*VM*; 0-1000 µg/mL) for 48 h. May-Grünwald/Giemsa staining (A) and immunofluorescence analysis for MyHC-II (B) were performed. Myotube diameters were measured using *Image J* software. WB analysis for MyHC-II was performed with tubulin used as loading control (C). Reported are representative images with indicated the percentage of myotube areas (A) or myotube diameters (µm; B). Data are means ± SEM (A,B) or SD (C) of three independent experiments. Statistical analysis was conducted using one-way ANOVA.

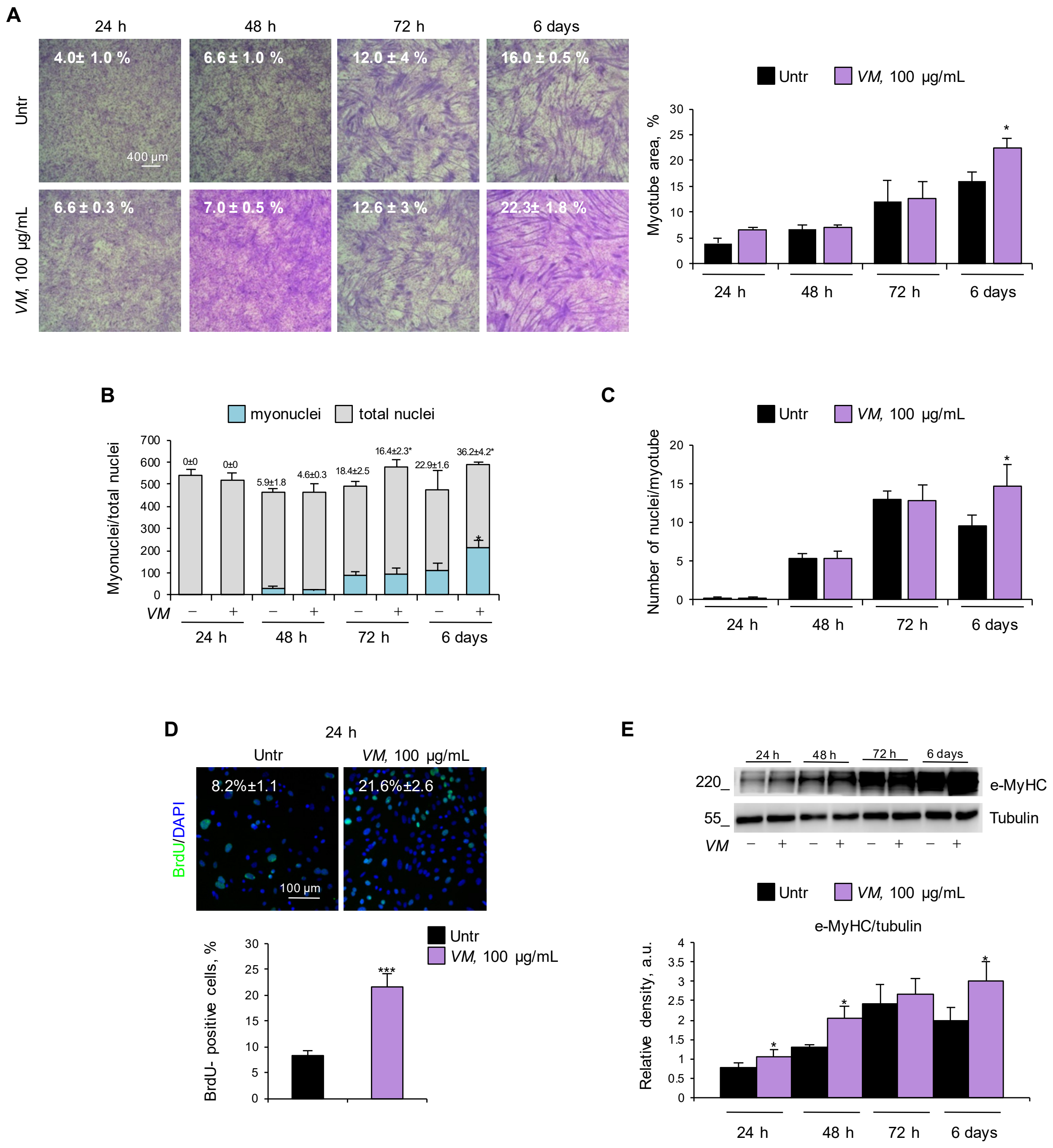

Figure S4. ***V. macrocarpon*** **increases myoblast proliferation, terminal differentiation and fusion into myotubes. (A-E)** C2C12 myoblasts cultivated in GM were switched to differentiation medium (DM) in the absence or presence of *V. macrocarpon* (100 µg/mL) for the indicated times. The myotube areas were measured usng *Image J* software after May-Grünwald/Giemsa staining and reported as percentages (A). The myonuclei (nuclei inside myotubes) and nuclei of unfused myoblasts were counted. The fusion indexes are reported on top of the corresponding bar (B). The average numbers of nuclei inside each myotube were evaluated (C). BrdU assay was performed to evaluate the percentages of proliferating cells. Reported are the merged images of BrdU (green) and DAPI (blue) used to counterstain nuclei (D). WB analysis was performed for embryonal isoform of myosin heavy chain (e-MyHC). Reported are the relative densities with respect to tubulin (E). Reported are representative images (A,D,E). Data are means ± SEM (A-D) or SD (E) of three independent experiments. Statistical analysis was conducted using one-way ANOVA. * $p<0.05$ and *** $p<0.001$, significantly different from untreated myoblasts (Untr).

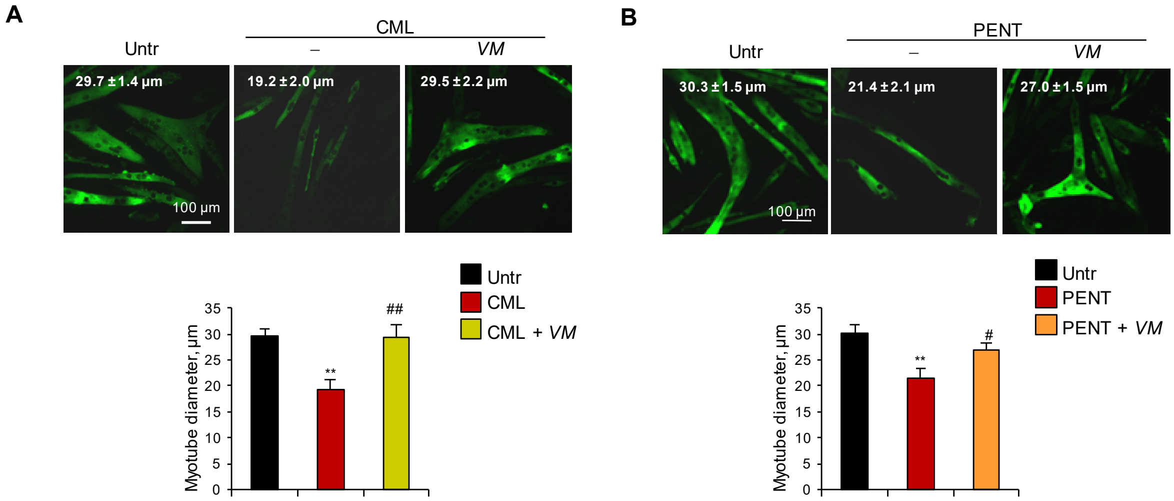

Figure S5. ***V. macrocarpon*** **counteracts CML- or PENT-induced myotube atrophy. (A,B)** C2C12 myotubes were treated with CML (400 µM) (A) or PENT (2 µM) (B) for 48 h in the absence or presence of *V. macrocarpon* (*VM*) (100 µg/mL). Immunofluorescence analysis for MyHC-II was performed and myotube diameters were measured using *Image J* software. The values of myotube diameters (µm) are reported. Reported are representative images. Data are the means±SEM of three independent experiments. Statistical analysis was conducted using one-way ANOVA. **$p<0.01$ significantly different from untreated (Untr). #$p<0.05$ and ##$p<0.01$, significantly different from CML or PENT.

A

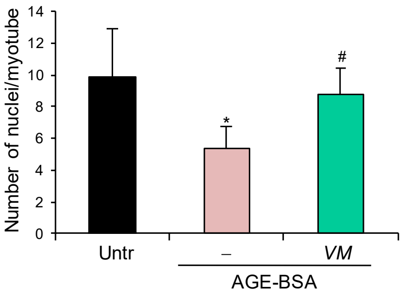

Fusion index (%)	
Untr.	18.1±4.6
AGE-BSA	10.5±1.7 *
AGE-BSA+*VM*	18.6±1.8 #

B

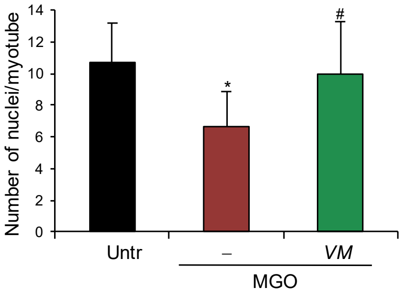

Fusion index (%)	
Untr.	19.9±2.4
MGO	13.5±4.1 *
MGO+*VM*	20.9±4.3 #

Figure S6. Diet-derived AGEs and AGE-BSA used different atrophying molecular mechanisms. (A,B) C2C12 myotubes were treated with AGE-BSA (400 µg/mL) (A) or MGO (500 µM) (0B) in the absence or presence of *VM* (100 µg/mL) for 48 h. The average of number of nuclei inside each myotube and the fusion index were calculated. Data are means ± SEM of three independent experiments. Statistical analysis was conducted using one-way ANOVA. * $p<0.05$ significantly different from untreated myotubes (Untr); # $p<0.05$, significantly different from AGE-BSA or MGO.

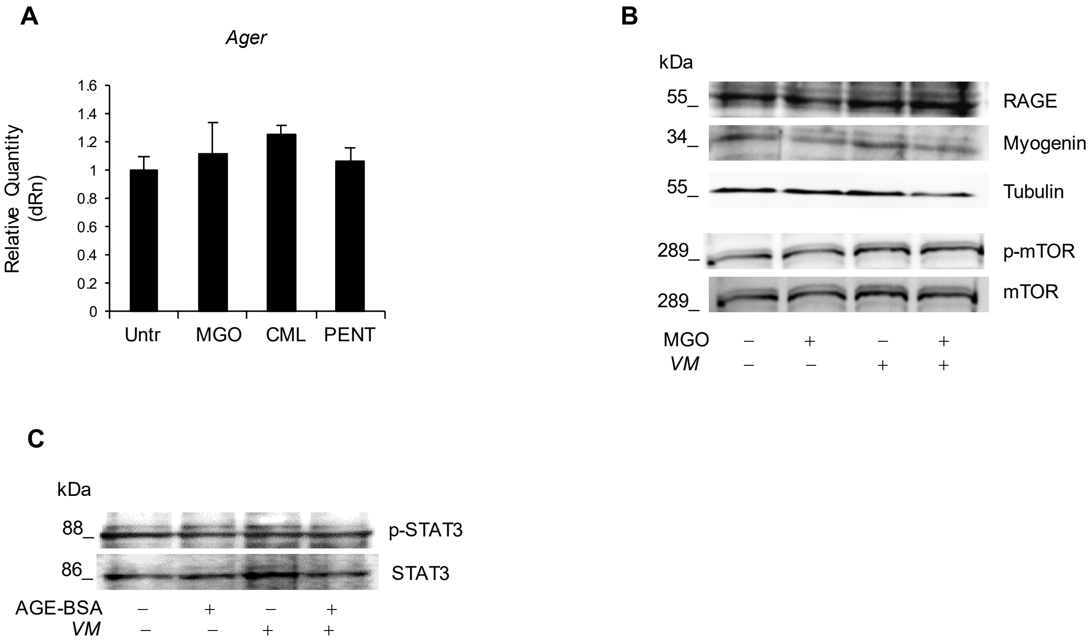

Figure S7. ***V. macrocarpon*** **preserves myotube growth in the presence of endogenous or exogenous AGEs**. (**A-C)** C2C12 myotubes were treated with MGO, CML, PENT or AGE-BSA for 24 h (A), 48 h (B) or 6 h (C). *Ager* (RAGE gene) levels were evaluated by real-time PCR by using *Gapdh* as the housekeeping gene (A). RAGE, myogenin, phosphorylated mTOR and STAT3 levels were analyzed by WB. Tubulin, total mTOR and STAT3 were used as loading control (B,C).Reported are representative images (B,C). Data are the means±SD of three independent experiments. Statistical analysis was conducted using one-way ANOVA.
